# Investigations on the occurrence of West Nile virus, Usutu virus and Sindbis virus RNA in avian louse flies (Diptera: Hippoboscidae) collected in Germany (2016–2022)

**DOI:** 10.1186/s13071-025-06841-9

**Published:** 2025-06-01

**Authors:** Markus Freick, Isabelle Vogt, Stephanie Schröter, Robert Kohl, Denise Heidl, Ruben Schreiter, Hein Sprong, Matthias Jentzsch

**Affiliations:** 1 Institute of Agricultural and Nutritional Sciences, Martin Luther University Halle-Wittenberg, 06120 Halle (Saale), Germany; 2 Faculty of Agriculture/Environment/Chemistry, HTW Dresden-University of Applied Sciences, Dresden, Germany; 3 Laboratory for Zoonoses and Environmental Microbiology, National Institute for Public Health and the Environment (RIVM), Bilthoven, The Netherlands

**Keywords:** Hippoboscidae, Avian louse flies, Arboviruses, Sentinel, Monitoring, West Nile virus, Usutu virus, Sindbis virus

## Abstract

**Background:**

As living vectors, arthropods play a crucial role in the transmission of viruses, bacteria and parasites. Previous research on virus transmission has focussed mainly on the roles of mosquitoes and ticks, while the potential importance of other blood-sucking arthropods such as louse flies (Hippoboscidae) has been somewhat neglected. The aim of this study was to detect viruses in avian louse flies from Germany to assess whether they could be used as sentinel organisms for monitoring arboviruses with zoonotic potential.

**Methods:**

We collected 1000 louse flies of the species *Crataerina hirundinis, C. pallida, Ornithomya avicularia, O. biloba, O. fringillina, O. chloropus, Ornithophila metallica* and *Pseudolynchia canariensis* in Germany and screened the samples via RT-PCR for West Nile virus (WNV), Usutu virus (USUV) and Sindbis virus (SINV), which are arboviruses with avian hosts as reservoirs.

**Results:**

While WNV was not detected, we found one louse fly positive for USUV and one for SINV RNA, both of which belonged to the species *O. avicularia* (*n* = 279). Therefore, the detection rates for both USUV and SINV were 0.1% (95% CI 0.0–0.3%) in the total sample and 0.36% (95% CI 0.00–1.09%) in *O. avicularia*. For the sample that tested positive for SINV, the PCR results were confirmed by sequencing a 288-bp segment that encoded part of the virus’s structural polyprotein.

**Conclusions:**

This is the first time that USUV RNA and SINV RNA have been detected in louse flies. In addition, it is the first detection of human pathogenic viruses in the louse fly species *O. avicularia*. The results of this study indicate that louse flies should not be neglected as possible sentinels of viral pathogens with zoonotic potential in the sense of the One Health concept.

**Graphical abstract:**

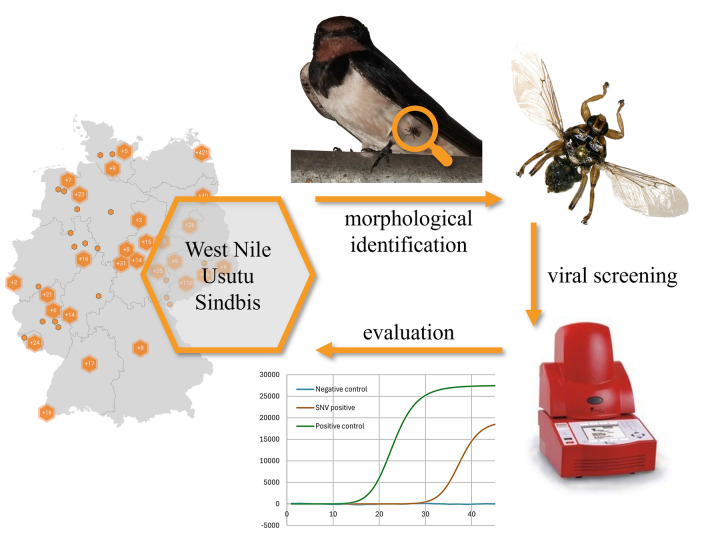

**Supplementary Information:**

The online version contains supplementary material available at 10.1186/s13071-025-06841-9.

## Background

Louse flies, known as keds, are insects within the order Diptera and the suborder Brachycera. To date, 216 species of louse fly have been described worldwide [1–4], 17 of them in Germany [5]. These blood-sucking ectoparasites infest mammals but are most frequently found on birds [6]. Some louse fly species have a broad host range (polyxenia), while others are restricted to a few (oligoxenia) or individual host species (monoxenia) [7–9]. Polyxene avian louse flies (e.g. *Ornithomya avicularia* and *O. fringillina*) parasitise many different bird species and can be transported hundreds of kilometres because of the migratory behaviour of their hosts and their high degree of intrinsic mobility [10, 11]. In addition, there are louse flies that have a limited host range but live in the vicinity of human dwellings and livestock or horse stables: *Crataerina hirundinis* (which parasitises House Martins), *C. pallida* (which parasitises Common Swifts) and *O*. *biloba* (which parasitises Barn Swallows) [7, 12, 13]. In rare cases, they accidentally migrate to other bird species; only occasionally can different louse fly species be found on the same host [14]. Furthermore, louse flies can change their host individuals, which could support the possible transmission of pathogens between the birds, but it is unknown how often such changes generally happen [11, 15].

As living vectors, arthropods play a crucial role in the transmission of viruses, bacteria and parasites that are pathogenic to birds and/or mammals, including humans [16]. Past research on the transmission of viruses has focused on the roles of various mosquito (Culicidae) and tick (Ixodidae) species, while the potential importance of other blood-sucking arthropods such as louse flies (Hippoboscidae) has been investigated less intensively in veterinary and medical entomology [17].

West Nile virus (WNV) (Orthoflavivirus nilense, family Flaviviridae) [18] replicates in avian host reservoirs and is transmitted by mosquitoes belonging to the genus *Culex*. Mammals such as horses and humans are dead-end hosts [19]. WNV was first detected in 1937 in the West Nile district of Uganda [20]. In Europe, it first appeared in France in the early 1960 s; infections in humans, horses and birds have mainly been reported in southern and southeastern European countries [21]. Between 2012 and 2021, 16 European Economic Area (EEA) countries reported WNV infections in humans and animals, and 3632 autochthonous cases of WNV infections in humans were reported [22]. In animal hosts, WNV infections were predominantly detected in horses and different bird species [23]. It is now one of the most widespread arthropod-borne (arbo)viruses worldwide [24].

A WNV-infected bird was found for the first time in Germany in August 2018 [25]; since then, WNV has been detected in various wild and domestic bird species [26, 27] and horses [28, 29]. In the summer of 2019, the first mosquito-borne human WNV infections were reported in Germany. In subsequent years, further infections occurred in the summer and autumn months in the eastern regions of the country [30]. Meanwhile, the virus has been detected several times in mosquitoes in the same area, including in a pool of overwintering females probably of the species *Culex pipiens* Linnaeus, 1758, proving that the virus overwinters in German mosquito populations [31, 32].

In recent years, several European countries have reported a notable increase in WNV cases in humans and animals, indicating a changing and expanding epidemiological pattern with some regions reporting earlier seasonal onset and higher case numbers than in previous years [22]. This trend underscores the growing public health relevance of WNV across Europe. In response, WNV and Usutu virus (USUV) are included in national and regional surveillance programmes, often comprising integrated approaches involving human, animal and entomological surveillance [22, 33]. These surveillance systems provide valuable data that support timely public health interventions and are essential for understanding the dynamics and geographic spread of these arboviruses.

WNV can be life-threatening to horses and can lead to West Nile fever or West Nile neuroinvasive disease in humans. However, most infections result in either no or mild, flu-like symptoms [34]. Due to WNV's relevance to equine health in Europe, the European College of Equine Internal Medicine recently published a consensus statement on Flaviviridae infections in horses, recommending WNV vaccination for horses in endemic areas [35].

Gancz et al. [36] and Farajollahi et al. [37], who detected WNV RNA in the louse fly species *Icosta americana* in North America, provided the first evidence that avian louse flies can also play a role in the transmission of this virus. In addition to mosquitoes and louse flies, stable flies (*Stomoxys calcitrans*) may also be involved in WNV transmission [38, 39].

Closely related to WNV is the USUV (*Orthoflavivirus usutuense*, family Flaviviridae) [18]. It is an arbovirus originating in Africa, transmitted by mosquitoes and detected in almost 100 wild bird species, which generally do not contract the disease [18, 40, 41]. However, very susceptible bird species are also known, e.g. blackbirds. Clinically, these birds often show apathy and disorders of the central nervous system and mass mortality of birds can occur [41]. USUV probably first appeared in Europe in 1996, as shown by retrospective studies from Italy [42]. It was also detected in Austria in 2001, in Hungary in 2005, in Switzerland in 2006, in Spain in 2006 and in Italy in 2009 [43]. In Germany, it was first found in mosquitoes in 2010 [44]. The following year, it caused massive bird mortality in the northern Upper Rhine Plain and the neighbouring Palatinate and Neckar Valley areas. Since then, there have been repeated localised or regional bird deaths, particularly among blackbirds and zoo birds and owls in aviaries [26, 27, 45, 46].

Current findings show that in recent years, USUV has spread throughout Germany from the Rhine-Main region [47]. The transmission of USUV to humans occurs sporadically in endemic areas, particularly in people with immunosuppression [48]. Symptoms like fever and rash can be observed, and complications in the central nervous system can arise [49, 50]. However, more recent studies have detected the virus in healthy blood donors in Germany, Austria, France and Italy [51, 52] and a few cases in people with neurological symptoms [41]. To date, studies on the detection of USUV in avian louse fly species are not available in the literature.

Sindbis virus (SINV) (genus *Alphavirus,* family Togaviridae) [18] is a mosquito-borne and bird-associated zoonotic virus widely distributed in Africa, Eurasia, Australia and New Zealand [53]. It is transmitted among birds (Passeriformes, Galliformes and Anseriformes) by mosquitoes of the genera *Culex, Culiseta, Aedes* and *Anopheles*, while humans are accidental dead-end hosts [53, 54]. SINV was initially discovered in Germany in 2009 in mosquito species in the Upper Rhine Valley [55]. Subsequently, it was found in a Hooded Crow (*Corvus cornix*) in Berlin 1 year later [56]. Since then, SINV has been repeatedly detected in mosquitoes of the genera *Anopheles* and *Culex* [55, 57, 58], with evidence of competence in German mosquito populations [54]. Although the virus is widespread, human infections have been reported mainly in northern Europe (Finland, Sweden and Russia) and South Africa [59, 60]. A recent study suggested that Sindbis virus genotype I (SINV-I), the causal agent of Sindbis fever outbreaks in humans, was introduced only once from central Africa to Sweden in the 1920 s, with subsequent circulation there. This was followed by two introductions of SINV-I from Sweden into Finland and Germany around the 1960 s and the 1970 s [61]. Symptoms of SINV infection in humans include joint inflammation and pain, fever, rash and fatigue [62]. SINV-specific antibodies were identified in blood donors from southwest Germany in 2010/2011 [63]. According to the available literature, there have been no attempts to detect SINV in louse flies.

This study aimed to determine whether WNV, USUV and SINV RNA can be detected in avian louse flies in Germany via RT-PCR. SINV, USUV and WNV were selected because of their high zoonotic potential, proven occurrence in the sampling area and use of birds as reservoir hosts. If avian louse flies are suitable sentinel organisms for spreading these viruses, their targeted collection from wild birds and the subsequent detection of the pathogens could contribute to establishing early detection and effective monitoring systems. Similar “early warning systems” involving the examination of mosquitoes or ticks have already been proposed and discussed in the literature [64–68].

## Methods

### Louse fly collection and identification

Bird ringers, city pigeon associations and wild bird rescue centres across Germany were contacted and asked to collect louse flies. Those who took part in the project transferred the louse flies into Twist-Top-Vials (2 ml, Sörensen) filled with 1.5 ml of a 70% ethanol solution prepared with distilled water and (ethanol eurodenaturiert ≥ 99%, TechniSolv, VWR Chemicals, Darmstadt, Germany); noted the host bird species, date of collection and location of the ringing site; and sent the samples by mail to the Dresden University of Applied Sciences (Dresden/Germany). All specimens were stored in ethanol in the dark at room temperature until analysed. Louse fly morphological identification was carried out in the laboratory based on the classification keys of Büttiker [7] and Oboňa et al. [69]. Host species were classified according to the official species list of birds in Germany [70].

### Viral RNA screening

RNA was extracted from completely homogenised flies using the blackPrep Tick DNA/RNA Kit (IST Innuscreen GmbH, Berlin, Germany), following the manufacturer's instructions. The RNA was eluted using 50 μl of elution buffer and analysed with a Qubit 4 Fluorometer (Life Technologies GmbH, Darmstadt, Germany) using the Qubit^™^ RNA IQ Assay. cDNA synthesis was performed with the RevertAid First Strand cDNA Synthesis Kit (Life Technologies GmbH, Darmstadt, Germany) as described in the manual. Before synthesis, the template RNA (5 µl) was supplemented with a random hexamer primer and water to a total volume of 12 µl and incubated at 65 °C for 5 min. Real-time PCR assays were performed with the innuMIX qPCR MasterMix Probe (IST Innuscreen GmbH, Berlin, Germany) in a total volume of 20 µl. Therefore, 5 µl of RNA, 0.9 µM of each primer and 0.25 µM of the probe were used. The primers were synthesised by Microsynth Seqlab, Balgach, Switzerland. The primer sequences and appropriate cycling conditions are mentioned in Table 1. Each run included negative and positive controls.

**Table 1:** Primers, probes and the appropriate PCR programmes used in this study for the detection of West Nile virus, Usutu virus and Sindbis virus in louse fly samples from Germany

Name of primers	Primer sequence (5′−3′)			Target-gene	Fragment size	References
	Denaturation	Annealing	Cycles			
	[°C/s]	[°C/s]				
WNV F	AGTAGTTCGCCTGTGTGAGC			5′-Untranslated region	118	[89]
WNV R	GCCCTCCTGGTTTCTTAGA					
WNV probe	FAM-AATCCTCACAAACACTACTAAGTTTGTCA-BHQ-1					
Conditions	95/15	55/45	40			
USUV F	CGTTCTCGACTTTGACTA			Nonstructural protein 1 gene	88	[44]
USUV R	GCTAGTAGTTCTTATGGA					
USUV probe	FAM-ACCGTCACAATCACTGAAGCAT-BHQ1					
Conditions	95/15	55/45	40			
Sind F	CACWCCAAATGACCATGC			Nonstructural protein 1 gene	134	[55]
Sind R	KGTGCTCGGAAWACATTC					
Sind probe	FAM-CAGAGCATTTTCGCATCTGGC-BHQ1					
Conditions	94/15	60/60	45			

### Sequence analysis

For sequencing. a 288-bp-long fragment of the structural polyprotein of SINV was amplified using a nested PCR (outer primer: SIN OF 3′-ATACGACMAAAGCGGAG CAG-5′ and SIN OR 3′-GAACCGTTACGACCCGTACT-5′; inner primer: SINV IF 3′-GATACTTTCTCCTC GCGAAATG-5′ and SINV IR 3′-CAAGTGCGGCGATTACAAGAC-5′). The PCR mixture consisted of Phusion high-fidelity PCR Mastermix and HF buffer (Life Technologies GmbH, Darmstadt, Germany), primers (a final concentration of 0.5 μM), water and 5 or 1 μl of cDNA/PCR product with a final volume of 25 μl (outer/inner PCR). The fragment was amplified with the following parameters: initial denaturation at 98 °C for 30 s, 40 cycles of denaturation at 98 °C for 10 s, annealing at 53/56 °C for 30 s and elongation at 72 °C for 30 s, before a final elongation at 72 °C for 10 min. The PCR products were analysed on a 1.5% agarose gel, and the appropriate band was isolated using the innuPREP DOUBLEpure Kit (IST Innuscreen GmbH, Berlin, Germany) and subsequently sequenced by Microsynth Seqlab, Balgach, Switzerland.

### Statistical analyses

Microsoft Excel (Version 2013, Microsoft Corp., Redmond, WA, USA) was used for data collection and processing. IBM SPSS Statistics (version 23, SPSS Inc., Chicago, IL, USA) was used for further statistical analyses. Due to the low detection rates of viral RNA, the results are only presented descriptively as the number and percentage of positive samples. The 95% confidence intervals of the detection rates were calculated using the bootstrapping method (*n* = 1000) [71, 72].

## Results

**Fig. 1:**
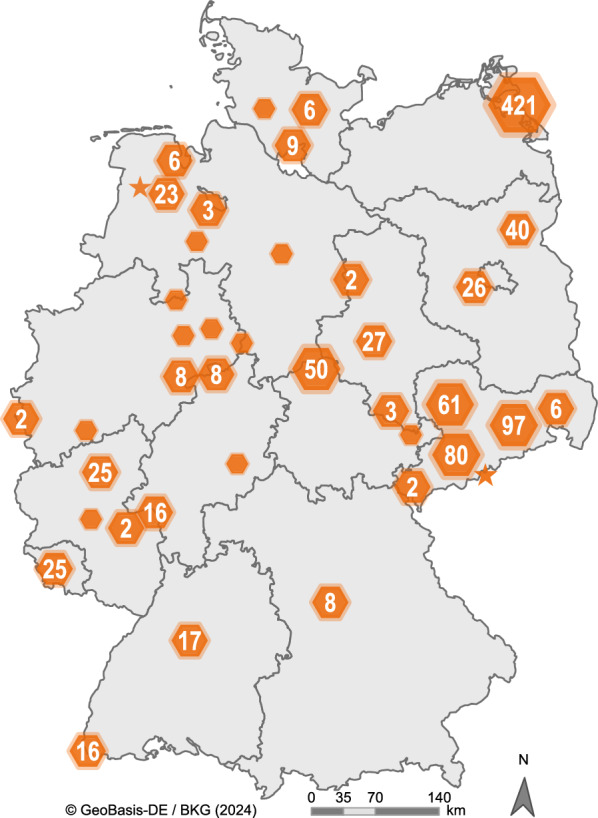
Visualisation of the sampling spots of the louse flies all over Germany. The map was created with ArcGIS Pro 10.5. The spots are graded by size in four groups: 1 (without number), 2–49, 50–149 and > 149 collected louse flies. The stars mark the location points of the louse flies that tested positive for USUV (Saxony) and SINV (Lower Saxony)

In total, 1000 specimens from eight louse fly species were examined. The louse flies were collected from 65 different avian host species in the federal states of Germany from 2016 to 2022 (Fig. 1; Table 2; Additional file 1: Table S1).

**Table 2:** Louse fly species with total numbers of samples, first description and type of host range, origin of host species, region of collection and years of sampling of the louse fly specimens examined in this study

Louse fly species^1^ (samples)	Type of host range	Origin from host species^2^ (*n*)	Origin from German Federal States^3^ (*n*)	Years of sampling
- First description				
*Crataerina hirundinis* (394)	Monoxenia	*Delichon urbicum* (393), *Hirundo rustica* (1)	HE (1), MV (374), NI (8), NW (1), RP (1), ST (9)	2018–2022
*-* Linnaeus, 1758				
*Crataerina pallida* (202)	Monoxenia	*Apus apus* (202)	BW (33), BY (8), BB (28), MV (5), SN (128)	2016–2021
- Latreille, 1812				
*Ornithomya avicularia* (279)	Polyxenia	*Accipiter gentilis* (1), *A. nisus* (6), *Acrocephalus arundinaceus* (8), *A*. *palustris* (1), *A*. *schoenobaenus* (1), *A*. *scirpaceus* (5), *Asio otus* (2), *Athene noctua* (1), *Bubo bubo* (6), *Buteo buteo* (1), *Carduelis carduelis* (2), *C. chloris* (1), *Coccothraustes coccothraustes* (4), *Coloeus monedula* (1), *Columba livia f*. *domestica* (3), *C. palumbus* (24), *Corvus cornix* (4), *C.* *corone* (8), *Cuculus canorus* (2), *D. urbicum* (1), *Dendrocopus major* (20), *Emberiza citrinella* (3), *Erithacus rubecula* (5), *Falco subbuteo* (1), *F. tinnunculus* (8), *Fringilla coelebs* (2), *Garullus glandarius* (8), *H. rustica* (1), *Jynx torquilla* (2), *Lanius collurio* (3), *Locustella luscinioides* (2), *Milvus milvus* (6), *Parus major* (3), *Passer domesticus* (1), *P. montanus* (2), *Phasianus colchicus* (1), *Phoenicurus ochruros* (2), *Phylloscopus collybita* (1), *P. trochilus* (1), *Pica pica* (12), *Picus viridis* (12), *Pyrrhula pyrrhula* (1), *Rallus aquaticus* (1), *Sitta europaea* (1), *Strix aluco* (3), *Sturnus vulgaris* (5), *Sylvia atricapilla* (6), *S. borin* (2), *S. nisoria* (1), *Turdus merula* (47), *T. philomelos* (18), *T. pilaris* (2), *T. viscivorus* (1), *Tyto alba* (1), unknown (12)	BB (26), HE (6), MV (20), NI (34), NW (19), RP (27), SL (25), SN (69), ST (15), SH (16), TH (22)	2018–2022
- Linnaeus, 1758				
*O. biloba* (55)	Monoxenia	*H. rustica* (52), *P. ochruros* (2), *R. aquaticus* (1)	BB (9), MV (16), RP (4), SN (23), ST (3)	2018–2022
- Dufour, 1827				
*O. chloropus* (2)	Polyxenia	*P. major* (1), *P. domesticus* (1)	MV (1), SN (1)	2020
- Bergroth, 1901				
*O. fringillina* (58)	Polyxenia	*A. palustris* (1), *A. scirpaceus* (6), *Cyanistes caeruleus* (2), *E. citrinella* (1), *E. rubecula* (10), *F. hypoleuca* (1), *L. collurio* (1), *Motacilla cinerea* (1), *P. major* (5), *P. collybita* (3), *P. trochilus* (1), *P. montanus* (2), *Poecile palustris* (2), *P. pyrrhula* (1), *Regulus ignicapilla* (3), *R. regulus* (1), *S. atricapilla* (13), *S. borin* (1), *T.* *philomelos* (1), unknown (2)	BB (3), MV (3), SN (27), ST (3), SH (2), TH (20)	2018–2022
- Curtis, 1863				
*O. metallica* (1)	Polyxenia	*A. scirpaceus* (1)	BB (1)	2021
- Schiner, 1864				
*P. canariensis* (9)	Oligoxenia	*C. livia f*. *domestica* (9)	HE (1), RP (8)	2021
- Macquart, 1839				

^1^*Crataerina pallida, Ornithomya avicularia, Ornithomya biloba, Ornithomya fringillina, Ornithophila metallica, Crataerina hirundinis, Pseudolynchia canariensis, Ornithomya chloropus*

^2^The avian species names are given alphabetically regardless of their systematic classification. They were taken from the official species list of birds in Germany [70]. The full species names are mentioned in the supplements (Additional file 1: Table S1)

^3^Origin from German Federal States: BW: Baden-Württemberg; BY: Bavaria; BB: Brandenburg; HE: Hesse; MV: Mecklenburg-Western Pomerania; NI: Lower Saxony; NW: North Rhine-Westphalia; RP: Rhineland-Palatinate; SL: Saarland; SN: Saxony; ST: Saxony-Anhalt; SH: Schleswig-Holstein; TH: Thuringia

WNV-specific RNA was not detected in any samples (0/1000; 0.0%; 95% confidence interval [CI]: 0.0–0.0%). However, we found one *Ornithomya avicularia* female that tested positive for USUV (sample ID JT-20-06; Ct value of 35.1; 1/1000; 0.1%; 95% CI 0.0–0.3%) and an additional female that tested positive for SINV (sample ID NB-21-19; mean Ct value of 31.7; 1/1000; 0.1%; 95% CI 0.0–0.3%). Among the 279 *O. avicularia* samples analysed, this corresponds to a detection rate of 0.36% (95% CI 0.00–1.09%) for both USUV and SINV within this louse fly species (Table 3).

**Table 3:** Detection rates of the hippoboscid 16S-rDNA gene as proof of successful nucleic acid isolation and of West Nile virus, Usutu virus and Sindbis virus RNA in the examined louse flies

Louse fly species	*n*	Detection rate (n/%)^1^		
		West Nile virus	Usutu virus	Sindbis virus
*Crataerina hirundinis*	394	0 (0.00)	0 (0.00)	0 (0.00)
*Crataerina pallida*	202	0 (0.00)	0 (0.00)	0 (0.00)
*Ornithomya avicularia*	279	0 (0.00)	**1 (0.36)**	**1 (0.36)**
*Ornithomya biloba*	55	0 (0.00)	0 (0.00)	0 (0.00)
*Ornithomya chloropus*	2	0 (0.00)	0 (0.00)	0 (0.00)
*Ornithomya fringillina*	58	0 (0.00)	0 (0.00)	0 (0.00)
*Ornithophila metallica*	1	0 (0.00)	0 (0.00)	0 (0.00)
*Pseudolynchia canariensis*	9	0 (0.00)	0 (0.00)	0 (0.00)
Total	1000	0 (0.00)	1 (0.10)	1 (0.10)

^1^Detection rates in relation to the number of individuals analysed within the louse fly species or the total number of samples (bold = positive samples)

**Fig. 2:**
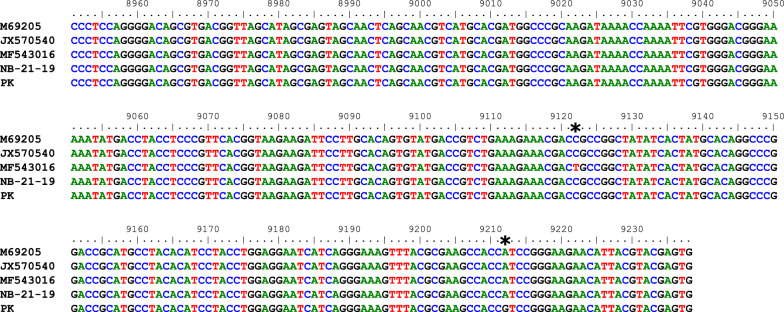
Alignment of the 288-bp fragment obtained from sequencing the SINV-positive sample NB-21–19 with the SINV reference strain Edsbyn (M69205), the two SINV isolates from Berlin [JX570540, (55)] and Gießen [MF543016, (52)] as well as the positive control used in this analysis (PK). Asteriks mark the SNPs

USUV RNA was found in an *O.* *avicularia* louse fly collected from a European Robin (*Erithacus rubecula*) in July 2020 in Saxony, while SINV RNA was detected in an *O.* *avicularia* specimen collected from a Common Wood Pigeon (*Columba palumbus*) in August 2021 in Lower Saxony (Additional file 1: Table S1). For the positive SINV sample, it was possible to sequence a 288-bp-long fragment [nucleotide (nt) position 8951 to 9238; positions are given according to the SINV reference strain Edsbyn, GenBank accession no. M69205] of the structural polyprotein. A comparison against the positive control yielded a single nucleotide polymorphism (SNP) at position 9212, whereby no SNP was observed compared to the reference strain (Fig. 2). Unfortunately, it was not possible to obtain sequencing data from the USUV-positive sample as it did not contain enough viral RNA.

## Discussion

This study aimed to detect WNV, USUV and SINV RNA in avian louse flies from Germany and assess whether they could be used as sentinel organisms for monitoring arboviruses with zoonotic potential.

Louse fly specimens were collected by expert bird ringers, city pigeon associations and wild bird rescue centres in a convenience sampling approach and brought to our laboratory. Eight species of louse flies with different numbers of individuals were identified. The species and sample number distribution were in line with expectations and consistent with the results of previous entomological projects [73–77]. Therefore, a typical sample of avian louse flies from Germany was represented in this study.

While mosquitoes and ticks are often the focus of research on vector-borne diseases [16, 78], louse flies have been largely ignored thus far in this context. This oversight can be attributed partly to their predominantly observed association with wild hosts, with infrequent identification on domestic animals and humans, particularly in the case of avian louse flies. However, it is known that avian louse flies can infest more than one bird host, and sometimes even two different species can be observed on a single host [11, 14, 15], increasing their potential as a vector for pathogens.

When louse flies are investigated for pathogens, researchers mainly focus on bacteria and/or parasites [17, 78–82]. However, two studies on WNV provided the first hint that louse flies can potentially play a role as vectors of viral pathogens. Gancz et al. [36] investigated an outbreak of WNV that caused many deaths in captive owls. Of the *I. americana* louse flies tested, 16/18 (88.9%) contained WNV RNA. Farajollahi et al. [37] used RT-PCR and detected WNV RNA in 4/85 (4.7%) louse flies of the species *I. americana*, considering both fully engorged and non-engorged specimens. For the reasons mentioned above, we have decided to elucidate the possible importance of avian louse flies as potential sentinels for the presence of WNV, USUV and SINV. These three viruses were selected because of their high zoonotic potential, proven occurrence in the sampling area and use of birds as reservoir hosts, as outlined before [26, 27, 41, 55, 57, 58, 62].

WNV RNA was not detected in any of the 1,000 louse flies examined in this study. This discrepancy with earlier studies with a remarkably smaller sample size, which detected WNV in 88.9 and 4.7% of louse flies [36, 37], could be explained by several factors. Both authors investigated the louse fly species *I. americana*, which is common in the southern Nearctic and relatively less abundant in the Neotropical region but not in Europe [83]. In addition, both studies used louse flies from pre-selected avian hosts, i.e. captive owls at an owl foundation in Canada with a high number of deaths during a WNV [36] or wild raptors delivered to a wildlife rehabilitation centre in the USA [37]. To the authors'knowledge, WNV has not yet been detected in European louse flies.

Two louse flies of the species *O. avicularia* were found to be positive for USUV or SINV RNA. This is the first detection of USUV and SINV gene fragments in louse flies worldwide. The genus *Ornithomya*, which belongs to the tribe Ornithomyini, comprises 32 species distributed globally, including one fossil species and four subspecies [1–4]. These species are characterised as fully winged bird parasites with highly specialised claws that facilitate adherence to and movement within their hosts'plumage [84]. In Europe, six species of *Ornithomya* have been identified thus far, with the most prevalent being *O. avicularia*, *O. fringillina* and *O. biloba*. *Ornithomya avicularia* has been documented in various hosts of different genera (i.e. it is a polyxene species) [85, 86]. The USUV-positive louse fly was collected from a European Robin (*Erithacus rubecula*) in July 2020 in Saxony. This bird species belongs to the order Passeriformes [70]. USUV has been detected many times in Europe and Germany in this group of birds [26, 27, 40, 45, 46, 87]. Moreover, SINV-RNA was detected in an *O*. *avicularia* specimen collected from a Common wood pigeon (*Columba palumbus*) in Lower Saxony. SINV was repeatedly detected in Germany in the context of mosquito monitoring programmes [55, 57, 58], with evidence of vector competence in mosquito populations [54]. SINV infections of wild birds have been reported in a Hooded Crow from Berlin [56] and, remarkably, a Common Wood Pigeon from Lower Saxony [53]. Both isolates were genetically distinct, suggesting the circulation of at least two different SINV strains in Germany.

A comparison of the SINV sequence obtained in this study with the two isolates from Berlin [56] and Giessen [53] shows more similarity to the Berlin isolate. However, this assumption is based on only one SNP (Fig. 2). Both Hooded Crows and Wood Pigeons are short-range migratory or particularly resident birds, which highlights the role of these birds in the regional maintenance of SINV. This is supported by detecting anti-SINV antibodies in different resident bird species in Germany [53]. Probably, the USUV- or SINV-RNA-positive *O. avicularia* specimens sucked blood from their viraemic avian hosts. Since the engorgement status of the louse flies was not determined in this study, the possible presence of viral RNA in an empty specimen cannot be excluded, as previously shown, at least for WNV [37].

In discussing the suitability of louse flies as sentinel organisms for selected arboviruses, a comparison with mosquito monitoring programmes seems to be useful. Scheuch et al. [57] screened 97,648 mosquitoes in 4144 pools collected throughout Germany from 2011 to 2016 for arboviruses. Two pools (0.05%) from Baden-Wuerttemberg and North Rhine-Westphalia tested positive for USUV, and three pools (0.07%) from Saxony-Anhalt and Berlin tested positive for SINV. The RT-qPCR Ct-values of the mosquitoes considered positive were mostly around 30 [57]. A more recent study screened 22,528 mosquitoes (2657 pools and 5107 single specimens) collected from 2019 to 2021 in Germany. WNV RNA was detected in 11 samples, USUV RNA in six samples, and SINV RNA in 21 samples. This corresponds with detection rates for WNV, USUV and SINV RNA of 0.0, 0.7 and 4.3% in 2019; 0.1, 0.1 and 0.1% in 2020 and 0.4, 0.1 and 0.5% in 2021, respectively. Samples containing USUV and WNV RNA consisted of mosquitoes collected in the East German Federal States of Brandenburg, Saxony and Saxony-Anhalt.

In contrast, samples containing SINV RNA originated from more widespread locations, including a sampling site in Lower Saxony [88]. This agrees with our results, as we found a USUV-positive louse fly in Saxony and a SINV-positive specimen in Lower Saxony. Compared to the detection rates of WNV, USUV and SINV in German mosquito monitoring programmes, we achieved percentages of positive louse fly samples in our study in the same ranges (0.0% for WNV and 0.36% for USUV and SINV each in *O. avicularia*). From an epidemiological and logistical point of view, however, mosquito surveillance programmes seem to be more suited to monitoring the presence and spread of arboviruses in a region, as they can be collected more easily using trap systems and thus generate significantly larger sample sizes. In contrast, avian louse flies must be collected manually from their hosts. Another indirect sentinel approach could be the serological screening of horses to detect antibodies against arboviruses, as has been proposed for the spread of flaviviruses in eastern and central Germany by Gothe et al. [29].

The present study detected viral RNA fragments in complete louse fly specimens via RT-PCR. Therefore, the data do not provide information on whether a viable virus, able to replicate and disseminate in the very louse fly species, was present or whether the louse fly would be able to transmit the virus. Future studies should therefore investigate the possible vector competence of avian louse flies experimentally. Furthermore, risk factors for virus detection in louse flies should be assessed using a representative sampling approach emphasising polyxene louse flies and considering the louse fly species, collection area, host species and engorgement status and sex of the louse flies.

## Conclusions

We could detect USUV and SINV RNA in louse flies for the first time to our knowledge. In addition, human pathogenic viruses were also detected for the first time to our knowledge in the louse fly species *O. avicularia*. Our results, as well as those from the studies of Gancz et al. [36] and Farajollahi et al. [37], indicate that avian louse flies should not be neglected as possible vectors of viral pathogens with zoonotic potential in the sense of the One Health concept. Since avian louse flies typically do not infest mammals, including humans [6], they may not pose a significant risk for the transmission of arboviruses to mammalian species; however, the polyxene species could contribute to the circulation of the virus in their avian reservoir host populations.

## Supplementary Information

Additional file 1: Table S1. Description of the individual samples in this study: Louse fly specieswith sample ID, sampling date, region of collection and federal stateas well as the host species.

## Data Availability

Data are provided within the manuscript or supplementary information files.
