# Supplementary material for: Investigations on the occurrence of West Nile virus, Usutu virus and Sindbis virus RNA in avian louse flies (Diptera: Hippoboscidae) collected in Germany (2016–2022)
Source: Parasit Vectors. 2025 Jun 1;18:200. doi: 10.1186/s13071-025-06841-9 (PMC12128526; doi:10.1186/s13071-025-06841-9)
Supplement: Supplementary file 1 — Additional file 1: Table S1. Description of the individual samples in this study: Louse fly specieswith sample ID, sampling date, region of collection and federal stateas well as the host species. [file 13071_2025_6841_MOESM1_ESM.docx]

**Supplementary information**

**Table S1** Description of the individual samples in this study: Louse fly species (Species) with sample ID, sampling date, region of collection and federal state (FS) as well as the host species

| **Sample** | **Species^1^** | **Date** | **Locality** | **FS^2^** | **Host species** |
| --- | --- | --- | --- | --- | --- |
| RM-R-20-01 | *C. pallida* | 06.07.2020 | Calw | BW | *Apus apus* |
| RM-R-20-02 | *C. pallida* | 06.07.2020 | Calw | BW | *Apus apus* |
| RM-R-20-03 | *C. pallida* | 06.07.2020 | Calw | BW | *Apus apus* |
| RM-R-20-04 | *C. pallida* | 06.07.2020 | Stammheim | BW | *Apus apus* |
| RM-R-20-05 | *C. pallida* | 06.07.2020 | Stammheim | BW | *Apus apus* |
| RM-R-21-01 | *C. pallida* | 06.07.2020 | Calw | BW | *Apus apus* |
| RM-R-21-02 | *C. pallida* | 06.07.2020 | Calw | BW | *Apus apus* |
| RM-R-21-03 | *C. pallida* | 06.07.2020 | Calw | BW | *Apus apus* |
| RM-R-21-04 | *C. pallida* | 06.07.2020 | Stammheim | BW | *Apus apus* |
| RM-R-21-05 | *C. pallida* | 06.07.2020 | Stammheim | BW | *Apus apus* |
| AW-R-20-01 | *C. pallida* | 08.07.2020 | Weil am Rhein | BW | *Apus apus* |
| AW-R-20-02 | *C. pallida* | 08.07.2020 | Weil am Rhein | BW | *Apus apus* |
| AW-R-20-03 | *C. pallida* | 08.07.2020 | Weil am Rhein | BW | *Apus apus* |
| AW-R-20-04 | *C. pallida* | 08.07.2020 | Weil am Rhein | BW | *Apus apus* |
| AW-R-20-05 | *C. pallida* | 08.07.2020 | Weil am Rhein | BW | *Apus apus* |
| AW-R-20-06 | *C. pallida* | 08.07.2020 | Weil am Rhein | BW | *Apus apus* |
| RM-R-20-06 | *C. pallida* | 09.07.2020 | Aidlingen | BW | *Apus apus* |
| RM-R-20-07 | *C. pallida* | 09.07.2020 | Aidlingen | BW | *Apus apus* |
| RM-R-20-08 | *C. pallida* | 09.07.2020 | Aidlingen | BW | *Apus apus* |
| RM-R-21-06 | *C. pallida* | 09.07.2020 | Aidlingen | BW | *Apus apus* |
| RM-R-21-07 | *C. pallida* | 09.07.2020 | Aidlingen | BW | *Apus apus* |
| RM-R-21-08 | *C. pallida* | 09.07.2020 | Aidlingen | BW | *Apus apus* |
| RM-R-21-09 | *C. pallida* | 09.07.2020 | Aidlingen | BW | *Apus apus* |
| AW-R-21-01 | *C. pallida* | 14.07.2021 | Weil am Rhein | BW | *Apus apus* |
| AW-R-21-02 | *C. pallida* | 14.07.2021 | Weil am Rhein | BW | *Apus apus* |
| AW-R-21-03 | *C. pallida* | 14.07.2021 | Weil am Rhein | BW | *Apus apus* |
| AW-R-21-04 | *C. pallida* | 14.07.2021 | Weil am Rhein | BW | *Apus apus* |
| AW-R-21-05 | *C. pallida* | 14.07.2021 | Weil am Rhein | BW | *Apus apus* |
| AW-R-21-06 | *C. pallida* | 14.07.2021 | Weil am Rhein | BW | *Apus apus* |
| AW-R-21-07 | *C. pallida* | 14.07.2021 | Weil am Rhein | BW | *Apus apus* |
| AW-R-21-08 | *C. pallida* | 14.07.2021 | Weil am Rhein | BW | *Apus apus* |
| AW-R-21-09 | *C. pallida* | 14.07.2021 | Weil am Rhein | BW | *Apus apus* |
| AW-R-21-10 | *C. pallida* | 14.07.2021 | Weil am Rhein | BW | *Apus apus* |
| KB-R-20-02 | *C. pallida* | 10.06.2020 | Schloss Ratibor, 91154 Roth | BY | *Apus apus* |
| KB-R-20-04 | *C. pallida* | 10.06.2020 | Schloss Ratibor, 91154 Roth | BY | *Apus apus* |
| KB-R-20-05 | *C. pallida* | 10.06.2020 | Schloss Ratibor, 91154 Roth | BY | *Apus apus* |
| KB-R-20-06 | *C. pallida* | 11.06.2020 | Gabrieliring 6, 91183 Abenberg | BY | *Apus apus* |
| KB-R-20-07 | *C. pallida* | 14.06.2020 | Evang. Kirche, 91154 Roth | BY | *Apus apus* |
| KB-R-20-08 | *C. pallida* | 14.06.2020 | Evang. Kirche, 91154 Roth | BY | *Apus apus* |
| KB-R-20-09 | *C. pallida* | 14.06.2020 | Evang. Kirche, 91154 Roth | BY | *Apus apus* |
| KB-R-20-10 | *C. pallida* | 14.06.2020 | Evang. Kirche, 91154 Roth | BY | *Apus apus* |
| RF-R-20-05 | *C. pallida* | 30.06.2020 | Groß Schönebeck (Barnim) | BB | *Apus apus* |
| RF-R-20-07 | *C. pallida* | 30.06.2020 | Groß Schönebeck (Barnim) | BB | *Apus apus* |
| SJ-R-20-01 | *C. pallida* | 06.07.2020 | Potsdam | BB | *Apus apus* |
| SJ-R-20-02 | *C. pallida* | 06.07.2020 | Potsdam | BB | *Apus apus* |
| SJ-R-20-03 | *C. pallida* | 06.07.2020 | Potsdam | BB | *Apus apus* |
| SJ-R-20-04 | *C. pallida* | 06.07.2020 | Potsdam | BB | *Apus apus* |
| SJ-R-21-01 | *C. pallida* | 06.07.2020 | Potsdam | BB | *Apus apus* |
| SJ-R-21-02 | *C. pallida* | 06.07.2020 | Potsdam | BB | *Apus apus* |
| SJ-R-21-03 | *C. pallida* | 06.07.2020 | Potsdam | BB | *Apus apus* |
| SJ-R-21-04 | *C. pallida* | 06.07.2020 | Potsdam | BB | *Apus apus* |
| SJ-R-21-05 | *C. pallida* | 06.07.2020 | Potsdam | BB | *Apus apus* |
| SJ-R-21-05 | *C. pallida* | 06.07.2020 | Potsdam | BB | *Apus apus* |
| SJ-R-21-06 | *C. pallida* | 06.07.2020 | Potsdam | BB | *Apus apus* |
| SJ-R-21-06 | *C. pallida* | 06.07.2020 | Potsdam | BB | *Apus apus* |
| SJ-R-21-07 | *C. pallida* | 06.07.2020 | Potsdam | BB | *Apus apus* |
| SJ-R-21-07 | *C. pallida* | 06.07.2020 | Potsdam | BB | *Apus apus* |
| SJ-R-21-08 | *C. pallida* | 06.07.2020 | Potsdam | BB | *Apus apus* |
| SJ-R-21-08 | *C. pallida* | 06.07.2020 | Potsdam | BB | *Apus apus* |
| SJ-R-21-09 | *C. pallida* | 06.07.2020 | Potsdam | BB | *Apus apus* |
| SJ-R-21-09 | *C. pallida* | 06.07.2020 | Potsdam | BB | *Apus apus* |
| SJ-R-21-10 | *C. pallida* | 06.07.2020 | Potsdam | BB | *Apus apus* |
| SJ-R-21-10 | *C. pallida* | 06.07.2020 | Potsdam | BB | *Apus apus* |
| RF-R-21-03 | *C. pallida* | 05.07.2021 | Groß Schönebeck (Barnim) | BB | *Apus apus* |
| RF-R-21-04 | *C. pallida* | 05.07.2021 | Groß Schönebeck (Barnim) | BB | *Apus apus* |
| RF-R-21-05 | *C. pallida* | 08.07.2021 | Joachimsthal | BB | *Apus apus* |
| RF-R-21-06 | *C. pallida* | 08.07.2021 | Groß Schönebeck (Barnim) | BB | *Apus apus* |
| RF-R-21-07 | *C. pallida* | 08.07.2021 | Groß Schönebeck (Barnim) | BB | *Apus apus* |
| RF-22-3 | *C. pallida* | 28.07.2022 | Joachimsthal | BB | *Apus apus* |
| RF-20-05 | *O. avicularia* | 21.07.2020 | Joachimsthal | BB | *Acrocephalus arundinaceus* |
| RF-20-25 | *O. avicularia* | 13.07.2021 | Joachimsthal | BB | *Acrocephalus arundinaceus* |
| RF-22-10 | *O. avicularia* | 11.07.2022 | Forst/Joachimsthal | BB | *Acrocephalus arundinaceus* |
| RF-22-7 | *O. avicularia* | 11.07.2022 | Forst/Joachimsthal | BB | *Acrocephalus arundinaceus* |
| RF-22-9 | *O. avicularia* | 11.07.2022 | Forst/Joachimsthal | BB | *Acrocephalus arundinaceus* |
| RF-22-12 | *O. avicularia* | 21.07.2022 | Forst/Joachimsthal | BB | *Acrocephalus arundinaceus* |
| WM-20-01 | *O. avicularia* | 11.07.2021 | Schiaßer See Tremsdorf | BB | *Acrocephalus schoenobaenus* |
| RF-20-08 | *O. avicularia* | 17.08.2020 | Joachimsthal | BB | *Acrocephalus scirpaceus* |
| RF-20-09 | *O. avicularia* | 25.08.2020 | Joachimsthal | BB | *Acrocephalus scirpaceus* |
| RF-20-11 | *O. avicularia* | 25.08.2020 | Joachimsthal | BB | *Acrocephalus scirpaceus* |
| RF-22-14 | *O. avicularia* | 11.08.2022 | Forst/Joachimsthal | BB | *Acrocephalus scirpaceus* |
| RF-22-8 | *O. avicularia* | 11.07.2022 | Forst/Joachimsthal | BB | *Erithacus rubecula* |
| RF-20-10 | *O. avicularia* | 25.08.2020 | Joachimsthal | BB | *Lanius collurio* |
| WM-20-02 | *O. avicularia* | 24.07.2021 | Schiaßer See Tremsdorf | BB | *Locustella luscinioides* |
| WM-20-03 | *O. avicularia* | 24.07.2021 | Schiaßer See Tremsdorf | BB | *Locustella luscinioides* |
| RF-20-04 | *O. avicularia* | 13.07.2020 | Joachimsthal | BB | *Parus major* |
| RF-22-13 | *O. avicularia* | 28.07.2022 | Joachimsthal | BB | *Phoenicurus ochruros* |
| RF-22-5 | *O. avicularia* | 09.07.2022 | Joachimsthal | BB | *Pica pica* |
| RF-22-6 | *O. avicularia* | 09.07.2022 | Joachimsthal | BB | *Pica pica* |
| RF-22-15 | *O. avicularia* | 17.08.2022 | Joachimsthal | BB | *Sturnus vulgaris* |
| RF-20-12 | *O. avicularia* | 25.08.2020 | Joachimsthal | BB | *Sylvia atricapilla* |
| RF-20-01 | *O. avicularia* | 14.05.2020 | Joachimsthal | BB | *Turdus merula* |
| RF-20-02 | *O. avicularia* | 24.06.2020 | Joachimsthal | BB | *Turdus merula* |
| RF-20-03 | *O. avicularia* | 07.07.2020 | Joachimsthal | BB | *Turdus merula* |
| RF-20-06 | *O. avicularia* | 31.07.2020 | Joachimsthal | BB | *Turdus philomelos* |
| RF-20-07 | *O. avicularia* | 31.07.2020 | Joachimsthal | BB | *Turdus philomelos* |
| TB-R-20-08 | *O. biloba* | 01.06.2020 | Wüstenhain | BB | *Hirundo rustica* |
| RF-R-20-01 | *O. biloba* | 05.06.2020 | Friedrichswalde (Barnim) | BB | *Hirundo rustica* |
| RF-R-20-02 | *O. biloba* | 05.06.2020 | Friedrichswalde (Barnim) | BB | *Hirundo rustica* |
| RF-R-20-03 | *O. biloba* | 05.06.2020 | Friedrichswalde (Barnim) | BB | *Hirundo rustica* |
| RF-R-20-04 | *O. biloba* | 05.06.2020 | Friedrichswalde (Barnim) | BB | *Hirundo rustica* |
| RF-R-21-01 | *O. biloba* | 29.06.2021 | Friedrichswalde (Barnim) | BB | *Hirundo rustica* |
| RF-22-11 | *O. biloba* | 18.07.2022 | Friedrichswalde | BB | *Hirundo rustica* |
| RF-22-16 | *O. biloba* | 01.09.2022 | Joachimsthal | BB | *Phoenicurus ochruros* |
| RF-22-16a | *O. biloba* | 01.09.2022 | Joachimsthal | BB | *Phoenicurus ochruros* |
| CG-R-20-15 | *O. fringillina* | 24.07.2021 | Schiaßer See Tremsdorf | BB | *Acrocephalus scirpaceus* |
| WM-20-05 | *O. fringillina* | 24.07.2021 | Schiaßer See Tremsdorf | BB | *Acrocephalus scirpaceus* |
| RF-20-13 | *O. fringillina* | 10.10.2020 | Grimnitzsee | BB | *Regulus regulus* |
| WM-20-04 | *O. metallica* | 24.07.2021 | Schiaßer See Tremsdorf | BB | *Acrocephalus scirpaceus* |
| AR-22-10 | *C. hirundinis* | 29.06.2022 | Blankenau | HE | *Hirundo rustica* |
| AR-22-16 | *O. avicularia* | 11.07.2022 | Wolfhagen | HE | *Bubo bubo* |
| FH-R-20-18 | *O. avicularia* | 22.06.2021 | Wiesbaden-Delkenheim | HE | *Columba palumbus* |
| FH-R-20-19 | *O. avicularia* | 08.07.2021 | Wiesbaden-Rheingauviertel | HE | *Columba palumbus* |
| FH-R-20-23 | *O. avicularia* | 10.07.2021 | Wiesbaden | HE | *Columba palumbus* |
| AR-22-6 | *O. avicularia* | 18.06.2022 | Niederlistingen | HE | *Milvus milvus* |
| AR-22-11 | *O. avicularia* | 03.07.2022 | Berndorf | HE | *Pica pica* |
| FH-R-20-31 | *P. canariensis* | 31.10.2021 | Oestrich-Winkel | HE | *Columba livia f*. *domestica* |
| GO-18-01 | *C. hirundinis* | 22.05.2018 | Greifswalder Oie | MV | *Delichon urbicum* |
| GO-18-03 | *C. hirundinis* | 24.05.2018 | Greifswalder Oie | MV | *Delichon urbicum* |
| GO-18-04 | *C. hirundinis* | 24.05.2018 | Greifswalder Oie | MV | *Delichon urbicum* |
| GO-18-06 | *C. hirundinis* | 26.05.2018 | Greifswalder Oie | MV | *Delichon urbicum* |
| GO-18-06a | *C. hirundinis* | 26.05.2018 | Greifswalder Oie | MV | *Delichon urbicum* |
| GO-18-06b | *C. hirundinis* | 26.05.2018 | Greifswalder Oie | MV | *Delichon urbicum* |
| GO-18-06c | *C. hirundinis* | 26.05.2018 | Greifswalder Oie | MV | *Delichon urbicum* |
| GO-18-06d | *C. hirundinis* | 26.05.2018 | Greifswalder Oie | MV | *Delichon urbicum* |
| GO-18-07 | *C. hirundinis* | 27.05.2018 | Greifswalder Oie | MV | *Delichon urbicum* |
| GO-18-07 | *C. hirundinis* | 27.05.2018 | Greifswalder Oie | MV | *Delichon urbicum* |
| GO-18-09 | *C. hirundinis* | 30.05.2018 | Greifswalder Oie | MV | *Delichon urbicum* |
| GO-18-09a | *C. hirundinis* | 30.05.2018 | Greifswalder Oie | MV | *Delichon urbicum* |
| GO-18-09b | *C. hirundinis* | 30.05.2018 | Greifswalder Oie | MV | *Delichon urbicum* |
| GO-18-12 | *C. hirundinis* | 06.06.2018 | Greifswalder Oie | MV | *Delichon urbicum* |
| GO-18-13 | *C. hirundinis* | 06.06.2018 | Greifswalder Oie | MV | *Delichon urbicum* |
| GO-18-15 | *C. hirundinis* | 06.06.2018 | Greifswalder Oie | MV | *Delichon urbicum* |
| GO-18-16 | *C. hirundinis* | 06.06.2018 | Greifswalder Oie | MV | *Delichon urbicum* |
| GO-18-17 | *C. hirundinis* | 06.06.2018 | Greifswalder Oie | MV | *Delichon urbicum* |
| GO-18-17a | *C. hirundinis* | 06.06.2018 | Greifswalder Oie | MV | *Delichon urbicum* |
| GO-18-17b | *C. hirundinis* | 06.06.2018 | Greifswalder Oie | MV | *Delichon urbicum* |
| GO-18-18 | *C. hirundinis* | 06.06.2018 | Greifswalder Oie | MV | *Delichon urbicum* |
| GO-18-18a | *C. hirundinis* | 06.06.2018 | Greifswalder Oie | MV | *Delichon urbicum* |
| GO-18-18b | *C. hirundinis* | 06.06.2018 | Greifswalder Oie | MV | *Delichon urbicum* |
| GO-18-18c | *C. hirundinis* | 06.06.2018 | Greifswalder Oie | MV | *Delichon urbicum* |
| GO-18-20 | *C. hirundinis* | 06.06.2018 | Greifswalder Oie | MV | *Delichon urbicum* |
| GO-18-20a | *C. hirundinis* | 06.06.2018 | Greifswalder Oie | MV | *Delichon urbicum* |
| GO-18-20b | *C. hirundinis* | 06.06.2018 | Greifswalder Oie | MV | *Delichon urbicum* |
| GO-18-20c | *C. hirundinis* | 06.06.2018 | Greifswalder Oie | MV | *Delichon urbicum* |
| GO-18-20d | *C. hirundinis* | 06.06.2018 | Greifswalder Oie | MV | *Delichon urbicum* |
| GO-18-21 | *C. hirundinis* | 06.06.2018 | Greifswalder Oie | MV | *Delichon urbicum* |
| GO-18-22 | *C. hirundinis* | 06.06.2018 | Greifswalder Oie | MV | *Delichon urbicum* |
| GO-18-26 | *C. hirundinis* | 07.06.2018 | Greifswalder Oie | MV | *Delichon urbicum* |
| GO-18-26a | *C. hirundinis* | 07.06.2018 | Greifswalder Oie | MV | *Delichon urbicum* |
| GO-18-26b | *C. hirundinis* | 07.06.2018 | Greifswalder Oie | MV | *Delichon urbicum* |
| GO-18-26c | *C. hirundinis* | 07.06.2018 | Greifswalder Oie | MV | *Delichon urbicum* |
| GO-18-26d | *C. hirundinis* | 07.06.2018 | Greifswalder Oie | MV | *Delichon urbicum* |
| GO-18-26e | *C. hirundinis* | 07.06.2018 | Greifswalder Oie | MV | *Delichon urbicum* |
| GO-18-27 | *C. hirundinis* | 07.06.2018 | Greifswalder Oie | MV | *Delichon urbicum* |
| GO-18-27a | *C. hirundinis* | 07.06.2018 | Greifswalder Oie | MV | *Delichon urbicum* |
| GO-18-27b | *C. hirundinis* | 07.06.2018 | Greifswalder Oie | MV | *Delichon urbicum* |
| GO-18-28 | *C. hirundinis* | 07.06.2018 | Greifswalder Oie | MV | *Delichon urbicum* |
| GO-18-28a | *C. hirundinis* | 07.06.2018 | Greifswalder Oie | MV | *Delichon urbicum* |
| GO-18-28b | *C. hirundinis* | 07.06.2018 | Greifswalder Oie | MV | *Delichon urbicum* |
| GO-18-28c | *C. hirundinis* | 07.06.2018 | Greifswalder Oie | MV | *Delichon urbicum* |
| GO-18-29d | *C. hirundinis* | 07.06.2018 | Greifswalder Oie | MV | *Delichon urbicum* |
| GO-18-29e | *C. hirundinis* | 07.06.2018 | Greifswalder Oie | MV | *Delichon urbicum* |
| GO-18-29f | *C. hirundinis* | 07.06.2018 | Greifswalder Oie | MV | *Delichon urbicum* |
| GO-18-30 | *C. hirundinis* | 07.06.2018 | Greifswalder Oie | MV | *Delichon urbicum* |
| GO-18-30a | *C. hirundinis* | 07.06.2018 | Greifswalder Oie | MV | *Delichon urbicum* |
| GO-18-30b | *C. hirundinis* | 07.06.2018 | Greifswalder Oie | MV | *Delichon urbicum* |
| GO-18-30c | *C. hirundinis* | 07.06.2018 | Greifswalder Oie | MV | *Delichon urbicum* |
| GO-18-30d | *C. hirundinis* | 07.06.2018 | Greifswalder Oie | MV | *Delichon urbicum* |
| GO-18-30e | *C. hirundinis* | 07.06.2018 | Greifswalder Oie | MV | *Delichon urbicum* |
| GO-18-30f | *C. hirundinis* | 07.06.2018 | Greifswalder Oie | MV | *Delichon urbicum* |
| GO-18-30g | *C. hirundinis* | 07.06.2018 | Greifswalder Oie | MV | *Delichon urbicum* |
| GO-18-31 | *C. hirundinis* | 07.06.2018 | Greifswalder Oie | MV | *Delichon urbicum* |
| GO-18-32 | *C. hirundinis* | 07.06.2018 | Greifswalder Oie | MV | *Delichon urbicum* |
| GO-18-32a | *C. hirundinis* | 07.06.2018 | Greifswalder Oie | MV | *Delichon urbicum* |
| GO-18-33 | *C. hirundinis* | 07.06.2018 | Greifswalder Oie | MV | *Delichon urbicum* |
| GO-18-34 | *C. hirundinis* | 07.06.2018 | Greifswalder Oie | MV | *Delichon urbicum* |
| GO-18-35 | *C. hirundinis* | 08.06.2018 | Greifswalder Oie | MV | *Delichon urbicum* |
| GO-18-35a | *C. hirundinis* | 08.06.2018 | Greifswalder Oie | MV | *Delichon urbicum* |
| GO-18-35b | *C. hirundinis* | 08.06.2018 | Greifswalder Oie | MV | *Delichon urbicum* |
| GO-18-35c | *C. hirundinis* | 08.06.2018 | Greifswalder Oie | MV | *Delichon urbicum* |
| GO-18-35d | *C. hirundinis* | 08.06.2018 | Greifswalder Oie | MV | *Delichon urbicum* |
| GO-18-37 | *C. hirundinis* | 08.06.2018 | Greifswalder Oie | MV | *Delichon urbicum* |
| GO-18-37a | *C. hirundinis* | 08.06.2018 | Greifswalder Oie | MV | *Delichon urbicum* |
| GO-18-38 | *C. hirundinis* | 09.06.2018 | Greifswalder Oie | MV | *Delichon urbicum* |
| GO-18-38a | *C. hirundinis* | 09.06.2018 | Greifswalder Oie | MV | *Delichon urbicum* |
| GO-18-39 | *C. hirundinis* | 09.06.2018 | Greifswalder Oie | MV | *Delichon urbicum* |
| GO-18-39a | *C. hirundinis* | 09.06.2018 | Greifswalder Oie | MV | *Delichon urbicum* |
| GO-18-39b | *C. hirundinis* | 09.06.2018 | Greifswalder Oie | MV | *Delichon urbicum* |
| GO-18-39c | *C. hirundinis* | 09.06.2018 | Greifswalder Oie | MV | *Delichon urbicum* |
| GO-18-40 | *C. hirundinis* | 27.06.2018 | Greifswalder Oie | MV | *Delichon urbicum* |
| GO-18-40a | *C. hirundinis* | 27.06.2018 | Greifswalder Oie | MV | *Delichon urbicum* |
| GO-18-40b | *C. hirundinis* | 27.06.2018 | Greifswalder Oie | MV | *Delichon urbicum* |
| GO-18-41 | *C. hirundinis* | 27.06.2018 | Greifswalder Oie | MV | *Delichon urbicum* |
| GO-18-42 | *C. hirundinis* | 27.06.2018 | Greifswalder Oie | MV | *Delichon urbicum* |
| GO-18-44 | *C. hirundinis* | 27.06.2018 | Greifswalder Oie | MV | *Delichon urbicum* |
| GO-18-45 | *C. hirundinis* | 27.06.2018 | Greifswalder Oie | MV | *Delichon urbicum* |
| GO-18-45a | *C. hirundinis* | 27.06.2018 | Greifswalder Oie | MV | *Delichon urbicum* |
| GO-18-46 | *C. hirundinis* | 27.06.2018 | Greifswalder Oie | MV | *Delichon urbicum* |
| GO-18-47 | *C. hirundinis* | 27.06.2018 | Greifswalder Oie | MV | *Delichon urbicum* |
| GO-18-48 | *C. hirundinis* | 27.06.2018 | Greifswalder Oie | MV | *Delichon urbicum* |
| GO-18-48a | *C. hirundinis* | 27.06.2018 | Greifswalder Oie | MV | *Delichon urbicum* |
| GO-18-48b | *C. hirundinis* | 27.06.2018 | Greifswalder Oie | MV | *Delichon urbicum* |
| GO-18-49c | *C. hirundinis* | 27.06.2018 | Greifswalder Oie | MV | *Delichon urbicum* |
| GO-18-49d | *C. hirundinis* | 27.06.2018 | Greifswalder Oie | MV | *Delichon urbicum* |
| GO-18-50 | *C. hirundinis* | 02.07.2018 | Greifswalder Oie | MV | *Delichon urbicum* |
| GO-18-50a | *C. hirundinis* | 02.07.2018 | Greifswalder Oie | MV | *Delichon urbicum* |
| GO-18-50b | *C. hirundinis* | 02.07.2018 | Greifswalder Oie | MV | *Delichon urbicum* |
| GO-18-50c | *C. hirundinis* | 02.07.2018 | Greifswalder Oie | MV | *Delichon urbicum* |
| GO-18-52 | *C. hirundinis* | 02.07.2018 | Greifswalder Oie | MV | *Delichon urbicum* |
| GO-18-53 | *C. hirundinis* | 02.07.2018 | Greifswalder Oie | MV | *Delichon urbicum* |
| GO-18-53a | *C. hirundinis* | 02.07.2018 | Greifswalder Oie | MV | *Delichon urbicum* |
| GO-18-54 | *C. hirundinis* | 02.07.2018 | Greifswalder Oie | MV | *Delichon urbicum* |
| GO-18-55 | *C. hirundinis* | 02.07.2018 | Greifswalder Oie | MV | *Delichon urbicum* |
| GO-18-57 | *C. hirundinis* | 04.07.2018 | Greifswalder Oie | MV | *Delichon urbicum* |
| GO-18-68 | *C. hirundinis* | 07.08.2018 | Greifswalder Oie | MV | *Delichon urbicum* |
| GO-18-69 | *C. hirundinis* | 07.08.2018 | Greifswalder Oie | MV | *Delichon urbicum* |
| GO-18-75 | *C. hirundinis* | 09.08.2018 | Greifswalder Oie | MV | *Delichon urbicum* |
| GO-18-77 | *C. hirundinis* | 11.08.2018 | Greifswalder Oie | MV | *Delichon urbicum* |
| GO-18-77a | *C. hirundinis* | 11.08.2018 | Greifswalder Oie | MV | *Delichon urbicum* |
| GO-18-77b | *C. hirundinis* | 11.08.2018 | Greifswalder Oie | MV | *Delichon urbicum* |
| GO-19-02 | *C. hirundinis* | 28.05.2019 | Greifswalder Oie | MV | *Delichon urbicum* |
| GO-19-03 | *C. hirundinis* | 28.05.2019 | Greifswalder Oie | MV | *Delichon urbicum* |
| GO-19-03a | *C. hirundinis* | 28.05.2019 | Greifswalder Oie | MV | *Delichon urbicum* |
| GO-19-03b | *C. hirundinis* | 28.05.2019 | Greifswalder Oie | MV | *Delichon urbicum* |
| GO-19-03c | *C. hirundinis* | 28.05.2019 | Greifswalder Oie | MV | *Delichon urbicum* |
| GO-19-04 | *C. hirundinis* | 28.05.2019 | Greifswalder Oie | MV | *Delichon urbicum* |
| GO-19-04a | *C. hirundinis* | 28.05.2019 | Greifswalder Oie | MV | *Delichon urbicum* |
| GO-19-04b | *C. hirundinis* | 28.05.2019 | Greifswalder Oie | MV | *Delichon urbicum* |
| GO-19-05 | *C. hirundinis* | 28.05.2019 | Greifswalder Oie | MV | *Delichon urbicum* |
| GO-19-05a | *C. hirundinis* | 28.05.2019 | Greifswalder Oie | MV | *Delichon urbicum* |
| GO-19-05b | *C. hirundinis* | 28.05.2019 | Greifswalder Oie | MV | *Delichon urbicum* |
| GO-19-05c | *C. hirundinis* | 28.05.2019 | Greifswalder Oie | MV | *Delichon urbicum* |
| GO-19-05d | *C. hirundinis* | 28.05.2019 | Greifswalder Oie | MV | *Delichon urbicum* |
| GO-19-05e | *C. hirundinis* | 28.05.2019 | Greifswalder Oie | MV | *Delichon urbicum* |
| GO-19-05f | *C. hirundinis* | 28.05.2019 | Greifswalder Oie | MV | *Delichon urbicum* |
| GO-19-05g | *C. hirundinis* | 28.05.2019 | Greifswalder Oie | MV | *Delichon urbicum* |
| GO-19-06 | *C. hirundinis* | 28.05.2019 | Greifswalder Oie | MV | *Delichon urbicum* |
| GO-19-06a | *C. hirundinis* | 28.05.2019 | Greifswalder Oie | MV | *Delichon urbicum* |
| GO-19-06b | *C. hirundinis* | 28.05.2019 | Greifswalder Oie | MV | *Delichon urbicum* |
| GO-19-06c | *C. hirundinis* | 28.05.2019 | Greifswalder Oie | MV | *Delichon urbicum* |
| GO-19-07 | *C. hirundinis* | 28.05.2019 | Greifswalder Oie | MV | *Delichon urbicum* |
| GO-19-08 | *C. hirundinis* | 28.05.2019 | Greifswalder Oie | MV | *Delichon urbicum* |
| GO-19-08a | *C. hirundinis* | 28.05.2019 | Greifswalder Oie | MV | *Delichon urbicum* |
| GO-19-09 | *C. hirundinis* | 28.05.2019 | Greifswalder Oie | MV | *Delichon urbicum* |
| GO-19-10 | *C. hirundinis* | 28.05.2019 | Greifswalder Oie | MV | *Delichon urbicum* |
| GO-19-10a | *C. hirundinis* | 28.05.2019 | Greifswalder Oie | MV | *Delichon urbicum* |
| GO-19-10b | *C. hirundinis* | 28.05.2019 | Greifswalder Oie | MV | *Delichon urbicum* |
| GO-19-11 | *C. hirundinis* | 28.05.2019 | Greifswalder Oie | MV | *Delichon urbicum* |
| GO-19-11a | *C. hirundinis* | 28.05.2019 | Greifswalder Oie | MV | *Delichon urbicum* |
| GO-19-12 | *C. hirundinis* | 28.05.2019 | Greifswalder Oie | MV | *Delichon urbicum* |
| GO-19-12a | *C. hirundinis* | 28.05.2019 | Greifswalder Oie | MV | *Delichon urbicum* |
| GO-19-12b | *C. hirundinis* | 28.05.2019 | Greifswalder Oie | MV | *Delichon urbicum* |
| GO-19-12c | *C. hirundinis* | 28.05.2019 | Greifswalder Oie | MV | *Delichon urbicum* |
| GO-19-12d | *C. hirundinis* | 28.05.2019 | Greifswalder Oie | MV | *Delichon urbicum* |
| GO-19-13 | *C. hirundinis* | 28.05.2019 | Greifswalder Oie | MV | *Delichon urbicum* |
| GO-19-13a | *C. hirundinis* | 28.05.2019 | Greifswalder Oie | MV | *Delichon urbicum* |
| GO-19-13b | *C. hirundinis* | 28.05.2019 | Greifswalder Oie | MV | *Delichon urbicum* |
| GO-19-13c | *C. hirundinis* | 28.05.2019 | Greifswalder Oie | MV | *Delichon urbicum* |
| GO-19-13d | *C. hirundinis* | 28.05.2019 | Greifswalder Oie | MV | *Delichon urbicum* |
| GO-19-14 | *C. hirundinis* | 28.05.2019 | Greifswalder Oie | MV | *Delichon urbicum* |
| GO-19-14a | *C. hirundinis* | 28.05.2019 | Greifswalder Oie | MV | *Delichon urbicum* |
| GO-19-14b | *C. hirundinis* | 28.05.2019 | Greifswalder Oie | MV | *Delichon urbicum* |
| GO-19-14c | *C. hirundinis* | 28.05.2019 | Greifswalder Oie | MV | *Delichon urbicum* |
| GO-19-14d | *C. hirundinis* | 28.05.2019 | Greifswalder Oie | MV | *Delichon urbicum* |
| GO-19-14e | *C. hirundinis* | 28.05.2019 | Greifswalder Oie | MV | *Delichon urbicum* |
| GO-19-14f | *C. hirundinis* | 28.05.2019 | Greifswalder Oie | MV | *Delichon urbicum* |
| GO-19-14g | *C. hirundinis* | 28.05.2019 | Greifswalder Oie | MV | *Delichon urbicum* |
| GO-19-15 | *C. hirundinis* | 28.05.2019 | Greifswalder Oie | MV | *Delichon urbicum* |
| GO-19-17 | *C. hirundinis* | 03.06.2019 | Greifswalder Oie | MV | *Delichon urbicum* |
| GO-19-17a | *C. hirundinis* | 03.06.2019 | Greifswalder Oie | MV | *Delichon urbicum* |
| GO-19-17b | *C. hirundinis* | 03.06.2019 | Greifswalder Oie | MV | *Delichon urbicum* |
| GO-19-17 | *C. hirundinis* | 03.06.2019 | Greifswalder Oie | MV | *Delichon urbicum* |
| GO-19-18 | *C. hirundinis* | 03.06.2019 | Greifswalder Oie | MV | *Delichon urbicum* |
| GO-19-18a | *C. hirundinis* | 03.06.2019 | Greifswalder Oie | MV | *Delichon urbicum* |
| GO-19-19 | *C. hirundinis* | 03.06.2019 | Greifswalder Oie | MV | *Delichon urbicum* |
| GO-19-19a | *C. hirundinis* | 03.06.2019 | Greifswalder Oie | MV | *Delichon urbicum* |
| GO-19-19b | *C. hirundinis* | 03.06.2019 | Greifswalder Oie | MV | *Delichon urbicum* |
| GO-19-19c | *C. hirundinis* | 03.06.2019 | Greifswalder Oie | MV | *Delichon urbicum* |
| GO-19-19d | *C. hirundinis* | 03.06.2019 | Greifswalder Oie | MV | *Delichon urbicum* |
| GO-19-19e | *C. hirundinis* | 03.06.2019 | Greifswalder Oie | MV | *Delichon urbicum* |
| GO-19-19f | *C. hirundinis* | 03.06.2019 | Greifswalder Oie | MV | *Delichon urbicum* |
| GO-19-19g | *C. hirundinis* | 03.06.2019 | Greifswalder Oie | MV | *Delichon urbicum* |
| GO-19-19h | *C. hirundinis* | 03.06.2019 | Greifswalder Oie | MV | *Delichon urbicum* |
| GO-19-20 | *C. hirundinis* | 03.06.2019 | Greifswalder Oie | MV | *Delichon urbicum* |
| GO-19-20a | *C. hirundinis* | 03.06.2019 | Greifswalder Oie | MV | *Delichon urbicum* |
| GO-19-20b | *C. hirundinis* | 03.06.2019 | Greifswalder Oie | MV | *Delichon urbicum* |
| GO-19-20c | *C. hirundinis* | 03.06.2019 | Greifswalder Oie | MV | *Delichon urbicum* |
| GO-19-20d | *C. hirundinis* | 03.06.2019 | Greifswalder Oie | MV | *Delichon urbicum* |
| GO-19-21 | *C. hirundinis* | 03.06.2019 | Greifswalder Oie | MV | *Delichon urbicum* |
| GO-19-22 | *C. hirundinis* | 03.06.2019 | Greifswalder Oie | MV | *Delichon urbicum* |
| GO-19-22a | *C. hirundinis* | 03.06.2019 | Greifswalder Oie | MV | *Delichon urbicum* |
| GO-19-22b | *C. hirundinis* | 03.06.2019 | Greifswalder Oie | MV | *Delichon urbicum* |
| GO-19-22c | *C. hirundinis* | 03.06.2019 | Greifswalder Oie | MV | *Delichon urbicum* |
| GO-19-22d | *C. hirundinis* | 03.06.2019 | Greifswalder Oie | MV | *Delichon urbicum* |
| GO-19-22e | *C. hirundinis* | 03.06.2019 | Greifswalder Oie | MV | *Delichon urbicum* |
| GO-19-22f | *C. hirundinis* | 03.06.2019 | Greifswalder Oie | MV | *Delichon urbicum* |
| GO-19-23 | *C. hirundinis* | 03.06.2019 | Greifswalder Oie | MV | *Delichon urbicum* |
| GO-19-23 | *C. hirundinis* | 03.06.2019 | Greifswalder Oie | MV | *Delichon urbicum* |
| GO-19-23a | *C. hirundinis* | 03.06.2019 | Greifswalder Oie | MV | *Delichon urbicum* |
| GO-19-23b | *C. hirundinis* | 03.06.2019 | Greifswalder Oie | MV | *Delichon urbicum* |
| GO-19-24 | *C. hirundinis* | 03.06.2019 | Greifswalder Oie | MV | *Delichon urbicum* |
| GO-19-24a | *C. hirundinis* | 03.06.2019 | Greifswalder Oie | MV | *Delichon urbicum* |
| GO-19-24b | *C. hirundinis* | 03.06.2019 | Greifswalder Oie | MV | *Delichon urbicum* |
| GO-19-25 | *C. hirundinis* | 03.06.2019 | Greifswalder Oie | MV | *Delichon urbicum* |
| GO-19-25a | *C. hirundinis* | 03.06.2019 | Greifswalder Oie | MV | *Delichon urbicum* |
| GO-19-25b | *C. hirundinis* | 03.06.2019 | Greifswalder Oie | MV | *Delichon urbicum* |
| GO-19-25c | *C. hirundinis* | 03.06.2019 | Greifswalder Oie | MV | *Delichon urbicum* |
| GO-19-26 | *C. hirundinis* | 03.06.2019 | Greifswalder Oie | MV | *Delichon urbicum* |
| GO-19-26a | *C. hirundinis* | 03.06.2019 | Greifswalder Oie | MV | *Delichon urbicum* |
| GO-19-26b | *C. hirundinis* | 03.06.2019 | Greifswalder Oie | MV | *Delichon urbicum* |
| GO-19-27 | *C. hirundinis* | 03.06.2019 | Greifswalder Oie | MV | *Delichon urbicum* |
| GO-19-27a | *C. hirundinis* | 03.06.2019 | Greifswalder Oie | MV | *Delichon urbicum* |
| GO-19-27b | *C. hirundinis* | 03.06.2019 | Greifswalder Oie | MV | *Delichon urbicum* |
| GO-19-27c | *C. hirundinis* | 03.06.2019 | Greifswalder Oie | MV | *Delichon urbicum* |
| GO-19-27d | *C. hirundinis* | 03.06.2019 | Greifswalder Oie | MV | *Delichon urbicum* |
| GO-19-27e | *C. hirundinis* | 03.06.2019 | Greifswalder Oie | MV | *Delichon urbicum* |
| GO-19-28 | *C. hirundinis* | 03.06.2019 | Greifswalder Oie | MV | *Delichon urbicum* |
| GO-19-28a | *C. hirundinis* | 03.06.2019 | Greifswalder Oie | MV | *Delichon urbicum* |
| GO-19-29 | *C. hirundinis* | 03.06.2019 | Greifswalder Oie | MV | *Delichon urbicum* |
| GO-19-29a | *C. hirundinis* | 03.06.2019 | Greifswalder Oie | MV | *Delichon urbicum* |
| GO-19-29b | *C. hirundinis* | 03.06.2019 | Greifswalder Oie | MV | *Delichon urbicum* |
| GO-19-29c | *C. hirundinis* | 03.06.2019 | Greifswalder Oie | MV | *Delichon urbicum* |
| GO-19-29d | *C. hirundinis* | 03.06.2019 | Greifswalder Oie | MV | *Delichon urbicum* |
| GO-19-29e | *C. hirundinis* | 03.06.2019 | Greifswalder Oie | MV | *Delichon urbicum* |
| GO-19-30 | *C. hirundinis* | 03.06.2019 | Greifswalder Oie | MV | *Delichon urbicum* |
| GO-19-30a | *C. hirundinis* | 03.06.2019 | Greifswalder Oie | MV | *Delichon urbicum* |
| GO-19-30b | *C. hirundinis* | 03.06.2019 | Greifswalder Oie | MV | *Delichon urbicum* |
| GO-19-30c | *C. hirundinis* | 03.06.2019 | Greifswalder Oie | MV | *Delichon urbicum* |
| GO-19-31 | *C. hirundinis* | 03.06.2019 | Greifswalder Oie | MV | *Delichon urbicum* |
| GO-19-32 | *C. hirundinis* | 03.06.2019 | Greifswalder Oie | MV | *Delichon urbicum* |
| GO-19-32a | *C. hirundinis* | 03.06.2019 | Greifswalder Oie | MV | *Delichon urbicum* |
| GO-19-33 | *C. hirundinis* | 03.06.2019 | Greifswalder Oie | MV | *Delichon urbicum* |
| GO-19-33a | *C. hirundinis* | 03.06.2019 | Greifswalder Oie | MV | *Delichon urbicum* |
| GO-19-34 | *C. hirundinis* | 03.06.2019 | Greifswalder Oie | MV | *Delichon urbicum* |
| GO-19-34a | *C. hirundinis* | 03.06.2019 | Greifswalder Oie | MV | *Delichon urbicum* |
| GO-19-35 | *C. hirundinis* | 03.06.2019 | Greifswalder Oie | MV | *Delichon urbicum* |
| GO-19-36 | *C. hirundinis* | 03.06.2019 | Greifswalder Oie | MV | *Delichon urbicum* |
| GO-19-36a | *C. hirundinis* | 03.06.2019 | Greifswalder Oie | MV | *Delichon urbicum* |
| GO-19-36b | *C. hirundinis* | 03.06.2019 | Greifswalder Oie | MV | *Delichon urbicum* |
| GO-19-36c | *C. hirundinis* | 03.06.2019 | Greifswalder Oie | MV | *Delichon urbicum* |
| GO-19-37 | *C. hirundinis* | 03.06.2019 | Greifswalder Oie | MV | *Delichon urbicum* |
| GO-19-37a | *C. hirundinis* | 03.06.2019 | Greifswalder Oie | MV | *Delichon urbicum* |
| GO-19-38 | *C. hirundinis* | 03.06.2019 | Greifswalder Oie | MV | *Delichon urbicum* |
| GO-19-38a | *C. hirundinis* | 03.06.2019 | Greifswalder Oie | MV | *Delichon urbicum* |
| GO-19-39 | *C. hirundinis* | 03.06.2019 | Greifswalder Oie | MV | *Delichon urbicum* |
| GO-19-39a | *C. hirundinis* | 03.06.2019 | Greifswalder Oie | MV | *Delichon urbicum* |
| GO-19-39b | *C. hirundinis* | 03.06.2019 | Greifswalder Oie | MV | *Delichon urbicum* |
| GO-19-40 | *C. hirundinis* | 03.06.2019 | Greifswalder Oie | MV | *Delichon urbicum* |
| GO-19-40a | *C. hirundinis* | 03.06.2019 | Greifswalder Oie | MV | *Delichon urbicum* |
| GO-19-41 | *C. hirundinis* | 03.06.2019 | Greifswalder Oie | MV | *Delichon urbicum* |
| GO-19-41a | *C. hirundinis* | 03.06.2019 | Greifswalder Oie | MV | *Delichon urbicum* |
| GO-19-42 | *C. hirundinis* | 03.06.2019 | Greifswalder Oie | MV | *Delichon urbicum* |
| GO-19-43 | *C. hirundinis* | 03.06.2019 | Greifswalder Oie | MV | *Delichon urbicum* |
| GO-19-43a | *C. hirundinis* | 03.06.2019 | Greifswalder Oie | MV | *Delichon urbicum* |
| GO-19-43b | *C. hirundinis* | 03.06.2019 | Greifswalder Oie | MV | *Delichon urbicum* |
| GO-19-43c | *C. hirundinis* | 03.06.2019 | Greifswalder Oie | MV | *Delichon urbicum* |
| GO-19-43d | *C. hirundinis* | 03.06.2019 | Greifswalder Oie | MV | *Delichon urbicum* |
| GO-19-43e | *C. hirundinis* | 03.06.2019 | Greifswalder Oie | MV | *Delichon urbicum* |
| GO-19-44 | *C. hirundinis* | 03.06.2019 | Greifswalder Oie | MV | *Delichon urbicum* |
| GO-19-45 | *C. hirundinis* | 03.06.2019 | Greifswalder Oie | MV | *Delichon urbicum* |
| GO-19-46 | *C. hirundinis* | 03.06.2019 | Greifswalder Oie | MV | *Delichon urbicum* |
| GO-19-46a | *C. hirundinis* | 03.06.2019 | Greifswalder Oie | MV | *Delichon urbicum* |
| GO-19-46b | *C. hirundinis* | 03.06.2019 | Greifswalder Oie | MV | *Delichon urbicum* |
| GO-19-46c | *C. hirundinis* | 03.06.2019 | Greifswalder Oie | MV | *Delichon urbicum* |
| GO-19-47 | *C. hirundinis* | 03.06.2019 | Greifswalder Oie | MV | *Delichon urbicum* |
| GO-19-47a | *C. hirundinis* | 03.06.2019 | Greifswalder Oie | MV | *Delichon urbicum* |
| GO-19-48 | *C. hirundinis* | 03.06.2019 | Greifswalder Oie | MV | *Delichon urbicum* |
| GO-19-48a | *C. hirundinis* | 03.06.2019 | Greifswalder Oie | MV | *Delichon urbicum* |
| GO-19-48b | *C. hirundinis* | 03.06.2019 | Greifswalder Oie | MV | *Delichon urbicum* |
| GO-19-48c | *C. hirundinis* | 03.06.2019 | Greifswalder Oie | MV | *Delichon urbicum* |
| GO-19-49 | *C. hirundinis* | 03.06.2019 | Greifswalder Oie | MV | *Delichon urbicum* |
| GO-19-50 | *C. hirundinis* | 03.06.2019 | Greifswalder Oie | MV | *Delichon urbicum* |
| GO-19-50a | *C. hirundinis* | 03.06.2019 | Greifswalder Oie | MV | *Delichon urbicum* |
| GO-19-50b | *C. hirundinis* | 03.06.2019 | Greifswalder Oie | MV | *Delichon urbicum* |
| GO-19-51 | *C. hirundinis* | 03.06.2019 | Greifswalder Oie | MV | *Delichon urbicum* |
| GO-19-51a | *C. hirundinis* | 03.06.2019 | Greifswalder Oie | MV | *Delichon urbicum* |
| GO-19-51b | *C. hirundinis* | 03.06.2019 | Greifswalder Oie | MV | *Delichon urbicum* |
| GO-19-51c | *C. hirundinis* | 03.06.2019 | Greifswalder Oie | MV | *Delichon urbicum* |
| GO-19-52 | *C. hirundinis* | 03.06.2019 | Greifswalder Oie | MV | *Delichon urbicum* |
| GO-19-52a | *C. hirundinis* | 03.06.2019 | Greifswalder Oie | MV | *Delichon urbicum* |
| GO-19-52b | *C. hirundinis* | 03.06.2019 | Greifswalder Oie | MV | *Delichon urbicum* |
| GO-19-52c | *C. hirundinis* | 03.06.2019 | Greifswalder Oie | MV | *Delichon urbicum* |
| GO-19-53 | *C. hirundinis* | 03.06.2019 | Greifswalder Oie | MV | *Delichon urbicum* |
| GO-19-53a | *C. hirundinis* | 03.06.2019 | Greifswalder Oie | MV | *Delichon urbicum* |
| GO-19-53b | *C. hirundinis* | 03.06.2019 | Greifswalder Oie | MV | *Delichon urbicum* |
| GO-19-53c | *C. hirundinis* | 03.06.2019 | Greifswalder Oie | MV | *Delichon urbicum* |
| GO-19-53d | *C. hirundinis* | 03.06.2019 | Greifswalder Oie | MV | *Delichon urbicum* |
| GO-19-54 | *C. hirundinis* | 03.06.2019 | Greifswalder Oie | MV | *Delichon urbicum* |
| GO-19-54a | *C. hirundinis* | 03.06.2019 | Greifswalder Oie | MV | *Delichon urbicum* |
| GO-19-55 | *C. hirundinis* | 03.06.2019 | Greifswalder Oie | MV | *Delichon urbicum* |
| GO-19-55a | *C. hirundinis* | 03.06.2019 | Greifswalder Oie | MV | *Delichon urbicum* |
| GO-19-55b | *C. hirundinis* | 03.06.2019 | Greifswalder Oie | MV | *Delichon urbicum* |
| GO-19-55c | *C. hirundinis* | 03.06.2019 | Greifswalder Oie | MV | *Delichon urbicum* |
| GO-19-55d | *C. hirundinis* | 03.06.2019 | Greifswalder Oie | MV | *Delichon urbicum* |
| GO-19-55e | *C. hirundinis* | 03.06.2019 | Greifswalder Oie | MV | *Delichon urbicum* |
| GO-19-55f | *C. hirundinis* | 03.06.2019 | Greifswalder Oie | MV | *Delichon urbicum* |
| GO-19-55g | *C. hirundinis* | 03.06.2019 | Greifswalder Oie | MV | *Delichon urbicum* |
| GO-19-56h | *C. hirundinis* | 03.06.2019 | Greifswalder Oie | MV | *Delichon urbicum* |
| GO-19-57 | *C. hirundinis* | 07.06.2019 | Greifswalder Oie | MV | *Delichon urbicum* |
| GO-19-57a | *C. hirundinis* | 07.06.2019 | Greifswalder Oie | MV | *Delichon urbicum* |
| GO-19-57b | *C. hirundinis* | 07.06.2019 | Greifswalder Oie | MV | *Delichon urbicum* |
| GO-19-57c | *C. hirundinis* | 07.06.2019 | Greifswalder Oie | MV | *Delichon urbicum* |
| GO-19-58 | *C. hirundinis* | 07.06.2019 | Greifswalder Oie | MV | *Delichon urbicum* |
| GO-19-58a | *C. hirundinis* | 07.06.2019 | Greifswalder Oie | MV | *Delichon urbicum* |
| GO-19-59 | *C. hirundinis* | 07.06.2019 | Greifswalder Oie | MV | *Delichon urbicum* |
| GO-19-59a | *C. hirundinis* | 07.06.2019 | Greifswalder Oie | MV | *Delichon urbicum* |
| GO-19-59b | *C. hirundinis* | 07.06.2019 | Greifswalder Oie | MV | *Delichon urbicum* |
| GO-19-59c | *C. hirundinis* | 07.06.2019 | Greifswalder Oie | MV | *Delichon urbicum* |
| GO-19-59d | *C. hirundinis* | 07.06.2019 | Greifswalder Oie | MV | *Delichon urbicum* |
| GO-19-60 | *C. hirundinis* | 07.06.2019 | Greifswalder Oie | MV | *Delichon urbicum* |
| GO-19-60a | *C. hirundinis* | 07.06.2019 | Greifswalder Oie | MV | *Delichon urbicum* |
| GO-19-62 | *C. hirundinis* | 07.06.2019 | Greifswalder Oie | MV | *Delichon urbicum* |
| GO-19-62a | *C. hirundinis* | 07.06.2019 | Greifswalder Oie | MV | *Delichon urbicum* |
| GO-19-62b | *C. hirundinis* | 07.06.2019 | Greifswalder Oie | MV | *Delichon urbicum* |
| GO-19-62c | *C. hirundinis* | 07.06.2019 | Greifswalder Oie | MV | *Delichon urbicum* |
| GO-19-62d | *C. hirundinis* | 07.06.2019 | Greifswalder Oie | MV | *Delichon urbicum* |
| GO-19-62e | *C. hirundinis* | 07.06.2019 | Greifswalder Oie | MV | *Delichon urbicum* |
| GO-19-62f | *C. hirundinis* | 07.06.2019 | Greifswalder Oie | MV | *Delichon urbicum* |
| GO-19-62g | *C. hirundinis* | 07.06.2019 | Greifswalder Oie | MV | *Delichon urbicum* |
| GO-19-62h | *C. hirundinis* | 07.06.2019 | Greifswalder Oie | MV | *Delichon urbicum* |
| GO-19-63 | *C. hirundinis* | 07.06.2019 | Greifswalder Oie | MV | *Delichon urbicum* |
| GO-19-64 | *C. hirundinis* | 07.06.2019 | Greifswalder Oie | MV | *Delichon urbicum* |
| GO-19-64a | *C. hirundinis* | 07.06.2019 | Greifswalder Oie | MV | *Delichon urbicum* |
| GO-19-64b | *C. hirundinis* | 07.06.2019 | Greifswalder Oie | MV | *Delichon urbicum* |
| GO-19-64c | *C. hirundinis* | 07.06.2019 | Greifswalder Oie | MV | *Delichon urbicum* |
| GO-19-64d | *C. hirundinis* | 07.06.2019 | Greifswalder Oie | MV | *Delichon urbicum* |
| GO-19-66 | *C. hirundinis* | 07.06.2019 | Greifswalder Oie | MV | *Delichon urbicum* |
| GO-19-66a | *C. hirundinis* | 07.06.2019 | Greifswalder Oie | MV | *Delichon urbicum* |
| GO-19-66b | *C. hirundinis* | 07.06.2019 | Greifswalder Oie | MV | *Delichon urbicum* |
| GO-19-66c | *C. hirundinis* | 07.06.2019 | Greifswalder Oie | MV | *Delichon urbicum* |
| GO-19-66d | *C. hirundinis* | 07.06.2019 | Greifswalder Oie | MV | *Delichon urbicum* |
| GO-19-66e | *C. hirundinis* | 07.06.2019 | Greifswalder Oie | MV | *Delichon urbicum* |
| GO-19-67 | *C. hirundinis* | 07.06.2019 | Greifswalder Oie | MV | *Delichon urbicum* |
| GO-19-67a | *C. hirundinis* | 07.06.2019 | Greifswalder Oie | MV | *Delichon urbicum* |
| GO-19-67b | *C. hirundinis* | 07.06.2019 | Greifswalder Oie | MV | *Delichon urbicum* |
| GO-19-67 | *C. hirundinis* | 07.06.2019 | Greifswalder Oie | MV | *Delichon urbicum* |
| GO-19-68 | *C. hirundinis* | 07.06.2019 | Greifswalder Oie | MV | *Delichon urbicum* |
| GO-19-68a | *C. hirundinis* | 07.06.2019 | Greifswalder Oie | MV | *Delichon urbicum* |
| GO-19-68b | *C. hirundinis* | 07.06.2019 | Greifswalder Oie | MV | *Delichon urbicum* |
| GO-19-69 | *C. hirundinis* | 07.06.2019 | Greifswalder Oie | MV | *Delichon urbicum* |
| GO-19-69a | *C. hirundinis* | 07.06.2019 | Greifswalder Oie | MV | *Delichon urbicum* |
| GO-19-69b | *C. hirundinis* | 07.06.2019 | Greifswalder Oie | MV | *Delichon urbicum* |
| GO-19-70 | *C. hirundinis* | 07.06.2019 | Greifswalder Oie | MV | *Delichon urbicum* |
| GO-19-70a | *C. hirundinis* | 07.06.2019 | Greifswalder Oie | MV | *Delichon urbicum* |
| GO-19-70b | *C. hirundinis* | 07.06.2019 | Greifswalder Oie | MV | *Delichon urbicum* |
| GO-19-71 | *C. hirundinis* | 07.06.2019 | Greifswalder Oie | MV | *Delichon urbicum* |
| GO-19-71a | *C. hirundinis* | 07.06.2019 | Greifswalder Oie | MV | *Delichon urbicum* |
| GO-19-71b | *C. hirundinis* | 07.06.2019 | Greifswalder Oie | MV | *Delichon urbicum* |
| GO-19-71c | *C. hirundinis* | 07.06.2019 | Greifswalder Oie | MV | *Delichon urbicum* |
| GO-19-71d | *C. hirundinis* | 07.06.2019 | Greifswalder Oie | MV | *Delichon urbicum* |
| GO-19-73 | *C. hirundinis* | 12.06.2019 | Greifswalder Oie | MV | *Delichon urbicum* |
| GO-19-74 | *C. hirundinis* | 12.06.2019 | Greifswalder Oie | MV | *Delichon urbicum* |
| GO-19-75 | *C. hirundinis* | 12.06.2019 | Greifswalder Oie | MV | *Delichon urbicum* |
| GO-19-76 | *C. hirundinis* | 12.06.2019 | Greifswalder Oie | MV | *Delichon urbicum* |
| GO-19-76a | *C. hirundinis* | 12.06.2019 | Greifswalder Oie | MV | *Delichon urbicum* |
| GO-19-76b | *C. hirundinis* | 12.06.2019 | Greifswalder Oie | MV | *Delichon urbicum* |
| GO-19-76c | *C. hirundinis* | 12.06.2019 | Greifswalder Oie | MV | *Delichon urbicum* |
| GO-19-76d | *C. hirundinis* | 12.06.2019 | Greifswalder Oie | MV | *Delichon urbicum* |
| GO-19-76e | *C. hirundinis* | 12.06.2019 | Greifswalder Oie | MV | *Delichon urbicum* |
| GO-19-78 | *C. hirundinis* | 20.06.2019 | Greifswalder Oie | MV | *Delichon urbicum* |
| GO-19-78a | *C. hirundinis* | 20.06.2019 | Greifswalder Oie | MV | *Delichon urbicum* |
| GO-19-78b | *C. hirundinis* | 20.06.2019 | Greifswalder Oie | MV | *Delichon urbicum* |
| GO-19-78c | *C. hirundinis* | 20.06.2019 | Greifswalder Oie | MV | *Delichon urbicum* |
| GO-19-78d | *C. hirundinis* | 20.06.2019 | Greifswalder Oie | MV | *Delichon urbicum* |
| GO-19-78e | *C. hirundinis* | 20.06.2019 | Greifswalder Oie | MV | *Delichon urbicum* |
| GO-19-78f | *C. hirundinis* | 20.06.2019 | Greifswalder Oie | MV | *Delichon urbicum* |
| GO-19-78g | *C. hirundinis* | 20.06.2019 | Greifswalder Oie | MV | *Delichon urbicum* |
| GO-19-78h | *C. hirundinis* | 20.06.2019 | Greifswalder Oie | MV | *Delichon urbicum* |
| GO-19-78i | *C. hirundinis* | 20.06.2019 | Greifswalder Oie | MV | *Delichon urbicum* |
| GO-19-78j | *C. hirundinis* | 20.06.2019 | Greifswalder Oie | MV | *Delichon urbicum* |
| GO-19-78k | *C. hirundinis* | 20.06.2019 | Greifswalder Oie | MV | *Delichon urbicum* |
| GO-19-79 | *C. hirundinis* | 11.07.2019 | Greifswalder Oie | MV | *Delichon urbicum* |
| GO-19-80 | *C. hirundinis* | 11.07.2019 | Greifswalder Oie | MV | *Delichon urbicum* |
| GO-19-81 | *C. hirundinis* | 11.07.2019 | Greifswalder Oie | MV | *Delichon urbicum* |
| GO-19-81a | *C. hirundinis* | 11.07.2019 | Greifswalder Oie | MV | *Delichon urbicum* |
| GO-19-81b | *C. hirundinis* | 11.07.2019 | Greifswalder Oie | MV | *Delichon urbicum* |
| GO-19-81c | *C. hirundinis* | 11.07.2019 | Greifswalder Oie | MV | *Delichon urbicum* |
| GO-19-81d | *C. hirundinis* | 11.07.2019 | Greifswalder Oie | MV | *Delichon urbicum* |
| GO-19-82 | *C. hirundinis* | 11.07.2019 | Greifswalder Oie | MV | *Delichon urbicum* |
| GO-19-83 | *C. hirundinis* | 11.07.2019 | Greifswalder Oie | MV | *Delichon urbicum* |
| GO-19-84 | *C. hirundinis* | 11.07.2019 | Greifswalder Oie | MV | *Delichon urbicum* |
| GO-19-84a | *C. hirundinis* | 11.07.2019 | Greifswalder Oie | MV | *Delichon urbicum* |
| GO-19-85 | *C. hirundinis* | 11.07.2019 | Greifswalder Oie | MV | *Delichon urbicum* |
| GO-19-85a | *C. hirundinis* | 11.07.2019 | Greifswalder Oie | MV | *Delichon urbicum* |
| GO-19-85b | *C. hirundinis* | 11.07.2019 | Greifswalder Oie | MV | *Delichon urbicum* |
| GO-19-85c | *C. hirundinis* | 11.07.2019 | Greifswalder Oie | MV | *Delichon urbicum* |
| GO-19-85d | *C. hirundinis* | 11.07.2019 | Greifswalder Oie | MV | *Delichon urbicum* |
| GO-19-85e | *C. hirundinis* | 11.07.2019 | Greifswalder Oie | MV | *Delichon urbicum* |
| GO-19-85f | *C. hirundinis* | 11.07.2019 | Greifswalder Oie | MV | *Delichon urbicum* |
| GO-19-85g | *C. hirundinis* | 11.07.2019 | Greifswalder Oie | MV | *Delichon urbicum* |
| RF-R-20-08 | *C. pallida* | 04.07.2020 | Wolgast | MV | *Apus apus* |
| RF-R-20-09 | *C. pallida* | 04.07.2020 | Wolgast | MV | *Apus apus* |
| RF-R-20-10 | *C. pallida* | 04.07.2020 | Wolgast | MV | *Apus apus* |
| RF-R-21-08 | *C. pallida* | 10.07.2021 | Wolgast | MV | *Apus apus* |
| RF-R-21-09 | *C. pallida* | 10.07.2021 | Wolgast | MV | *Apus apus* |
| GO-18-86 | *O. avicularia* | 07.09.2018 | Greifswalder Oie | MV | *Accipiter nisus* |
| GO-18-87 | *O. avicularia* | 14.09.2018 | Greifswalder Oie | MV | *Accipiter nisus* |
| GO-19-88 | *O. avicularia* | 01.08.2019 | Greifswalder Oie | MV | *Accipiter nisus* |
| GO-20-7 | *O. avicularia* | 22.05.2020 | Greifswalder Oie | MV | *Accipiter nisus* |
| GO-18-71 | *O. avicularia* | 07.08.2018 | Greifswalder Oie | MV | *Columba palumbus* |
| GO-19-89 | *O. avicularia* | 02.08.2019 | Greifswalder Oie | MV | *Corvus cornix* |
| GO-19-89a | *O. avicularia* | 02.08.2019 | Greifswalder Oie | MV | *Corvus cornix* |
| GO-19-89b | *O. avicularia* | 02.08.2019 | Greifswalder Oie | MV | *Corvus cornix* |
| GO-19-90 | *O. avicularia* | 02.08.2019 | Greifswalder Oie | MV | *Corvus cornix* |
| GO-19-92 | *O. avicularia* | 06.08.2019 | Greifswalder Oie | MV | *Cuculus canorus* |
| GO-18-82 | *O. avicularia* | 21.08.2018 | Greifswalder Oie | MV | *Phylloscopus trochilus* |
| GO-18-79 | *O. avicularia* | 13.08.2018 | Greifswalder Oie | MV | *Rallus aquaticus* |
| GO-20-6 | *O. avicularia* | 15.08.2020 | Greifswalder Oie | MV | *Sturnus vulgaris* |
| GO-20-1 | *O. avicularia* | 29.05.2020 | Greifswalder Oie | MV | *Sylvia atricapilla* |
| GO-18-05 | *O. avicularia* | 26.05.2018 | Greifswalder Oie | MV | *Turdus merula* |
| GO-18-10 | *O. avicularia* | 03.06.2018 | Greifswalder Oie | MV | *Turdus merula* |
| GO-19-16 | *O. avicularia* | 30.05.2019 | Greifswalder Oie | MV | *Turdus merula* |
| GO-20-2 | *O. avicularia* | 09.06.2020 | Greifswalder Oie | MV | *Turdus merula* |
| GO-18-84 | *O. avicularia* | 03.09.2018 | Greifswalder Oie | MV | *Turdus philomelos* |
| GO-18-65 | *O. avicularia* | no date | Greifswalder Oie | MV | *unknown* |
| GO-18-02 | *O. biloba* | 23.05.2018 | Greifswalder Oie | MV | *Hirundo rustica* |
| GO-18-24 | *O. biloba* | 07.06.2018 | Greifswalder Oie | MV | *Hirundo rustica* |
| GO-18-63 | *O. biloba* | 03.08.2018 | Greifswalder Oie | MV | *Hirundo rustica* |
| GO-18-74 | *O. biloba* | 09.08.2018 | Greifswalder Oie | MV | *Hirundo rustica* |
| GO-18-81 | *O. biloba* | 24.08.2018 | Greifswalder Oie | MV | *Hirundo rustica* |
| GO-18-81a | *O. biloba* | 24.08.2018 | Greifswalder Oie | MV | *Hirundo rustica* |
| GO-18-81b | *O. biloba* | 24.08.2018 | Greifswalder Oie | MV | *Hirundo rustica* |
| GO-18-90 | *O. biloba* | 24.08.2018 | Greifswalder Oie | MV | *Hirundo rustica* |
| GO-18-90 | *O. biloba* | 24.08.2018 | Greifswalder Oie | MV | *Hirundo rustica* |
| GO-18-90a | *O. biloba* | 24.08.2018 | Greifswalder Oie | MV | *Hirundo rustica* |
| GO-18-90b | *O. biloba* | 24.08.2018 | Greifswalder Oie | MV | *Hirundo rustica* |
| GO-19-61 | *O. biloba* | 07.06.2019 | Greifswalder Oie | MV | *Hirundo rustica* |
| GO-19-61 | *O. biloba* | 07.06.2019 | Greifswalder Oie | MV | *Hirundo rustica* |
| GO-19-61a | *O. biloba* | 07.06.2019 | Greifswalder Oie | MV | *Hirundo rustica* |
| GO-19-61b | *O. biloba* | 07.06.2019 | Greifswalder Oie | MV | *Hirundo rustica* |
| GO-20-3 | *O. biloba* | 09.06.2020 | Greifswalder Oie | MV | *Rallus aquaticus* |
| GO-20-4 | *O. chloropus* | 19.06.2020 | Greifswalder Oie | MV | *Passer domesticus* |
| GO-19-95 | *O. fringillina* | 29.08.2019 | Greifswalder Oie | MV | *Ficedula hypoleuca* |
| GO-19-91 | *O. fringillina* | 03.08.2019 | Greifswalder Oie | MV | *Parus major* |
| GO-19-93 | *O. fringillina* | 13.08.2019 | Greifswalder Oie | MV | *Phylloscopus trochilus* |
| CP-R-20-10 | *C. hirundinis* | 09.06.2020 | Oderhaus | NI | *Delichon urbicum* |
| CP-R-20-01 | *C. hirundinis* | 20.07.2020 | Oderhaus | NI | *Delichon urbicum* |
| CP-R-20-02 | *C. hirundinis* | 20.07.2020 | Oderhaus | NI | *Delichon urbicum* |
| CP-R-20-03 | *C. hirundinis* | 20.07.2020 | Oderhaus | NI | *Delichon urbicum* |
| CP-R-20-04 | *C. hirundinis* | 20.07.2020 | Oderhaus | NI | *Delichon urbicum* |
| CP-R-20-05 | *C. hirundinis* | 20.07.2020 | Oderhaus | NI | *Delichon urbicum* |
| CP-R-20-06 | *C. hirundinis* | 20.07.2020 | Oderhaus | NI | *Delichon urbicum* |
| CP-R-20-07 | *C. hirundinis* | 20.07.2020 | Oderhaus | NI | *Delichon urbicum* |
| NB-21-13 | *O. avicularia* | 03.08.2021 | Oldenburg | NI | *Accipiter gentilis* |
| NB-21-04 | *O. avicularia* | 29.06.2021 | Rastede | NI | *Asio otus* |
| NB-21-10 | *O. avicularia* | 12.07.2021 | Bassum | NI | *Asio otus* |
| AK-20-11 | *O. avicularia* | 08.07.2022 | Oldenburg, Nadorsterstraße | NI | *Buteo buteo* |
| AK-20-3 | *O. avicularia* | 01.07.2021 | Oldenburg, Bhf Süd | NI | *Columba palumbus* |
| AK-20-4 | *O. avicularia* | 01.07.2021 | Oldenburg, Bhf Süd | NI | *Columba palumbus* |
| AK-20-5 | *O. avicularia* | 01.07.2021 | Oldenburg, Bhf Nord | NI | *Columba palumbus* |
| AK-20-6 | *O. avicularia* | 04.07.2021 | Oldenburg, Parkhaus Galeria | NI | *Columba palumbus* |
| AK-20-7 | *O. avicularia* | 17.07.2021 | Oldenburg, Peterstraße | NI | *Columba palumbus* |
| AK-20-8 | *O. avicularia* | 18.08.2021 | Edewecht, Friedrich-Gehner-Straße | NI | *Columba palumbus* |
| AK-20-9 | *O. avicularia* | 20.08.2021 | Oldenburg, Innenstadt | NI | *Columba palumbus* |
| NB-21-19 | *O. avicularia* | 20.08.2021 | Oldenburg | NI | *Columba palumbus* |
| AK-20-10 | *O. avicularia* | 23.05.2022 | Rastede, Parkstraße | NI | *Columba palumbus* |
| NB-22-14 | *O. avicularia* | 02.07.2022 | Oldenburg | NI | *Columba palumbus* |
| AK-20-12 | *O. avicularia* | 10.07.2022 | Oldenburg-Donnerschwee | NI | *Columba palumbus* |
| NB-22-15 | *O. avicularia* | 14.07.2022 | Rastede | NI | *Columba palumbus* |
| NB-21-14 | *O. avicularia* | 10.08.2021 | Varel | NI | *Corvus corone* |
| NB-21-15 | *O. avicularia* | 10.08.2021 | Varel | NI | *Corvus corone* |
| NB-21-17 | *O. avicularia* | 15.08.2021 | Wiefelstedte | NI | *Corvus corone* |
| NB-21-12 | *O. avicularia* | 30.07.2021 | Ovelgönne | NI | *Cuculus canorus* |
| NB-22-05 | *O. avicularia* | 23.06.2022 | Barßel | NI | *Dendrocopos major* |
| NB-22-10 | *O. avicularia* | 27.06.2022 | Rastede | NI | *Dendrocopos major* |
| NB-21-05 | *O. avicularia* | 04.07.2021 | Eydelstedt | NI | *Falco subbuteo* |
| NB-22-19 | *O. avicularia* | 21.07.2022 | Friedeburg | NI | *Garrulus glandarius* |
| NB-22-12 | *O. avicularia* | 27.06.2022 | Oldenburg | NI | *Pica pica* |
| NB-21-11 | *O. avicularia* | 21.07.2021 | Rastede | NI | *Picus viridis* |
| NB-22-13 | *O. avicularia* | 30.06.2022 | Delmenhorst | NI | *Picus viridis* |
| NB-22-18 | *O. avicularia* | 20.07.2022 | Wiefelstede | NI | *Picus viridis* |
| NB-21-02 | *O. avicularia* | 26.06.2021 | Bockhorn | NI | *Sturnus vulgaris* |
| NB-21-01 | *O. avicularia* | 26.06.2021 | Oldenburg | NI | *Turdus merula* |
| NB-21-08 | *O. avicularia* | 10.07.2021 | Oldenburg | NI | *Turdus merula* |
| NB-22-11 | *O. avicularia* | 27.06.2022 | Delmenhorst | NI | *Turdus merula* |
| NB-22-20 | *O. avicularia* | 22.07.2022 | Wilhelmshaven | NI | *Turdus merula* |
| NB-22-7 | *O. avicularia* | 25.06.2022 | Zetel | NI | *Tyto alba* |
| AR-22-2 | *C. hirundinis* | 09.06.2022 | Haaren | NW | *Delichon urbicum* |
| MF-20-22 | *O. avicularia* | 07.08.2021 | Bonn | NW | *Accipiter nisus* |
| AR-21-7 | *O. avicularia* | 15.08.2021 | Bestwig | NW | *Bubo bubo* |
| AR-21-8 | *O. avicularia* | 22.08.2021 | Olsberg | NW | *Bubo bubo* |
| AR-21-9 | *O. avicularia* | 23.08.2021 | Warburg | NW | *Bubo bubo* |
| AR-21-11 | *O. avicularia* | 28.08.2021 | Olsberg | NW | *Bubo bubo* |
| AR-21-11a | *O. avicularia* | 28.08.2021 | Olsberg | NW | *Bubo bubo* |
| AR-21-10 | *O. avicularia* | 27.08.2021 | Padberg | NW | *Carduelis carduelis* |
| AR-21-5 | *O. avicularia* | 11.07.2021 | Brilon-Alme | NW | *Coccothraustes coccothraustes* |
| AR-22-7 | *O. avicularia* | 19.06.2022 | Fürstenberg | NW | *Coccothraustes coccothraustes* |
| AR-22-4 | *O. avicularia* | 14.06.2022 | Brilon | NW | *Coloeus monedula* |
| AR-22-3 | *O. avicularia* | 12.06.2022 | Brilon | NW | *Dendrocopos major* |
| AR-21-4 | *O. avicularia* | 02.07.2021 | Willebadessen | NW | *Falco tinnunculus* |
| AR-22-9 | *O. avicularia* | 29.06.2022 | Obermarsberg | NW | *Falco tinnunculus* |
| AR-22-12 | *O. avicularia* | 03.07.2022 | Aachen | NW | *Garrulus glandarius* |
| AR-22-8 | *O. avicularia* | 21.06.2022 | Borgholzhausen | NW | *Pica pica* |
| AR-21-3 | *O. avicularia* | 26.06.2021 | Marsberg-Westheim | NW | *Turdus merula* |
| AR-21-6 | *O. avicularia* | 29.07.2021 | Marsberg-Giershagen | NW | *Turdus pilaris* |
| AR-22-13 | *O. avicularia* | 04.07.2022 | Brilon | NW | *Turdus pilaris* |
| AR-22-1 | *O. avicularia* | 08.06.2022 | Westenholz | NW | *Turdus viscivorus* |
| MF-20-15 | *C. hirundinis* | 19.07.2021 | Kirchwald Eifel | RP | *Delichon urbicum* |
| MF-20-20 | *O. avicularia* | 30.07.2021 | Weibern | RP | *Accipiter nisus* |
| RF-22-2 | *O. avicularia* | 22.07.2022 | Hauptstraße 32, Mörsfeld | RP | *Columba livia f*. *domestica* |
| FH-R-20-24 | *O. avicularia* | no date | Mainz | RP | *Columba livia f*. *domestica* |
| FH-R-20-02 | *O. avicularia* | 30.06.2021 | Bodenheim, Kapellenstraße | RP | *Columba palumbus* |
| FH-R-20-03 | *O. avicularia* | 05.07.2021 | Essenheim in RheinHEn | RP | *Columba palumbus* |
| FH-R-20-22 | *O. avicularia* | 16.07.2021 | Mainz | RP | *Columba palumbus* |
| MF-20-19 | *O. avicularia* | 21.07.2021 | Mayen | RP | *Columba palumbus* |
| MF-20-19a | *O. avicularia* | 21.07.2021 | Mayen | RP | *Columba palumbus* |
| MF-20-23 | *O. avicularia* | 31.08.2021 | St. Goar | RP | *Columba palumbus* |
| MF-20-21 | *O. avicularia* | 30.07.2021 | Weibern | RP | *Delichon urbicum* |
| MF-20-02 | *O. avicularia* | 20.06.2021 | Breitenau | RP | *Dendrocopos major* |
| MF-20-03 | *O. avicularia* | 25.06.2021 | Breitenau | RP | *Dendrocopos major* |
| MF-20-04 | *O. avicularia* | 25.06.2021 | Breitenau | RP | *Dendrocopos major* |
| MF-20-05 | *O. avicularia* | 25.06.2021 | Breitenau | RP | *Dendrocopos major* |
| MF-20-07 | *O. avicularia* | 26.06.2021 | Kurtscheid | RP | *Dendrocopos major* |
| MF-20-08 | *O. avicularia* | 26.06.2021 | Langscheid Nettetal | RP | *Dendrocopos major* |
| MF-20-08a | *O. avicularia* | 26.06.2021 | Langscheid Nettetal | RP | *Dendrocopos major* |
| MF-20-08b | *O. avicularia* | 26.06.2021 | Langscheid Nettetal | RP | *Dendrocopos major* |
| MF-20-08c | *O. avicularia* | 26.06.2021 | Langscheid Nettetal | RP | *Dendrocopos major* |
| MF-20-08d | *O. avicularia* | 26.06.2021 | Langscheid Nettetal | RP | *Dendrocopos major* |
| MF-20-09 | *O. avicularia* | 26.06.2021 | Mayen | RP | *Garrulus glandarius* |
| MF-20-14 | *O. avicularia* | 16.07.2021 | Lederbach Eifel | RP | *Milvus milvus* |
| MF-20-06 | *O. avicularia* | 25.06.2021 | Andernach | RP | *Picus viridis* |
| MF-20-16 | *O. avicularia* | 20.07.2021 | Ney Hunsrück | RP | *Strix aluco* |
| MF-20-17 | *O. avicularia* | 20.07.2021 | Ney Hunsrück | RP | *Strix aluco* |
| MF-20-18 | *O. avicularia* | 20.07.2021 | Ney Hunsrück | RP | *Strix aluco* |
| MF-20-01 | *O. avicularia* | 18.06.2021 | Gutenberg | RP | *Sturnus vulgaris* |
| MF-20-11 | *O. biloba* | 27.06.2021 | Königsfeld Eifel | RP | *Hirundo rustica* |
| MF-20-11a | *O. biloba* | 27.06.2021 | Königsfeld Eifel | RP | *Hirundo rustica* |
| MF-20-11b | *O. biloba* | 27.06.2021 | Königsfeld Eifel | RP | *Hirundo rustica* |
| MF-20-24 | *O. biloba* | 02.09.2021 | Weitersbach | RP | *Hirundo rustica* |
| FH-R-20-17 | *P. canariensis* | 16.04.2021 | Mainz Hbf | RP | *Columba livia f*. *domestica* |
| FH-R-20-01 | *P. canariensis* | 27.05.2021 | Mainz Hbf | RP | *Columba livia f*. *domestica* |
| FH-R-20-06 | *P. canariensis* | 27.05.2021 | Mainz Hbf | RP | *Columba livia f*. *domestica* |
| FH-R-20-07 | *P. canariensis* | 27.05.2021 | Mainz Hbf | RP | *Columba livia f*. *domestica* |
| FH-R-20-08 | *P. canariensis* | 27.05.2021 | Mainz Hbf | RP | *Columba livia f*. *domestica* |
| FH-R-20-09 | *P. canariensis* | 27.05.2021 | Mainz Hbf | RP | *Columba livia f*. *domestica* |
| FH-R-20-21 | *P. canariensis* | 12.10.2021 | Mainz | RP | *Columba livia f*. *domestica* |
| FH-R-20-25 | *P. canariensis* | 13.10.2021 | Mainz | RP | *Columba livia f*. *domestica* |
| SV-22-13 | *O. avicularia* | 05.07.2022 | Saarlouis | SL | *Chloris chloris* |
| SV-22-7 | *O. avicularia* | 29.06.2022 | Quierschied | SL | *Corvus corone* |
| SV-22-7a | *O. avicularia* | 29.06.2022 | Quierschied | SL | *Corvus corone* |
| SV-22-8 | *O. avicularia* | 29.06.2022 | Saarbrücken | SL | *Corvus corone* |
| SV-22-8a | *O. avicularia* | 29.06.2022 | Saarbrücken | SL | *Corvus corone* |
| SV-22-16 | *O. avicularia* | 09.07.2022 | Merzig | SL | *Corvus corone* |
| SV-22-6 | *O. avicularia* | 28.06.2022 | Nalbach | SL | *Garrulus glandarius* |
| SV-22-6a | *O. avicularia* | 28.06.2022 | Nalbach | SL | *Garrulus glandarius* |
| SV-22-6b | *O. avicularia* | 28.06.2022 | Nalbach | SL | *Garrulus glandarius* |
| SV-22-11 | *O. avicularia* | 04.07.2022 | Völklingen | SL | *Garrulus glandarius* |
| SV-22-3 | *O. avicularia* | 20.06.2022 | Völklingen | SL | *Pica pica* |
| SV-22-15 | *O. avicularia* | 08.07.2022 | Saarbrücken | SL | *Pica pica* |
| SV-22-17 | *O. avicularia* | 09.07.2022 | Schmelz | SL | *Pica pica* |
| SV-22-18 | *O. avicularia* | 10.07.2022 | Schmelz | SL | *Pica pica* |
| SV-22-18 | *O. avicularia* | 10.07.2022 | Schmelz | SL | *Pica pica* |
| SV-22-18a | *O. avicularia* | 10.07.2022 | Schmelz | SL | *Pica pica* |
| SV-22-20 | *O. avicularia* | 15.07.2022 | Beckingen | SL | *Pica pica* |
| SV-22-5 | *O. avicularia* | 27.06.2022 | Saarbrücken | SL | *Picus viridis* |
| SV-22-10 | *O. avicularia* | 04.07.2022 | Saarbrücken | SL | *Picus viridis* |
| SV-22-19 | *O. avicularia* | 11.07.2022 | Beckingen | SL | *Picus viridis* |
| SV-22-19a | *O. avicularia* | 11.07.2022 | Beckingen | SL | *Picus viridis* |
| SV-22-19b | *O. avicularia* | 11.07.2022 | Beckingen | SL | *Picus viridis* |
| SV-22-19c | *O. avicularia* | 11.07.2022 | Beckingen | SL | *Picus viridis* |
| SV-22-19d | *O. avicularia* | 11.07.2022 | Beckingen | SL | *Picus viridis* |
| SV-22-19e | *O. avicularia* | 11.07.2022 | Beckingen | SL | *Picus viridis* |
| 10 | *C. pallida* | 01.07.2016 | Freital | SN | *Apus apus* |
| 11 | *C. pallida* | 01.07.2016 | Freital | SN | *Apus apus* |
| 12 | *C. pallida* | 03.07.2016 | Plauen | SN | *Apus apus* |
| 13 | *C. pallida* | 06.07.2016 | Dresden | SN | *Apus apus* |
| 14 | *C. pallida* | 06.07.2016 | Dresden | SN | *Apus apus* |
| 15 | *C. pallida* | 06.07.2016 | Dresden | SN | *Apus apus* |
| 16 | *C. pallida* | 06.07.2016 | Dresden | SN | *Apus apus* |
| 17 | *C. pallida* | 06.07.2016 | Dresden | SN | *Apus apus* |
| 18 | *C. pallida* | 06.07.2016 | Dresden | SN | *Apus apus* |
| 19 | *C. pallida* | 06.07.2016 | Dresden | SN | *Apus apus* |
| 20 | *C. pallida* | 06.07.2016 | Dresden | SN | *Apus apus* |
| 21 | *C. pallida* | 11.07.2016 | Dresden | SN | *Apus apus* |
| 22 | *C. pallida* | 11.07.2016 | Dresden | SN | *Apus apus* |
| 23 | *C. pallida* | 11.07.2016 | Dresden | SN | *Apus apus* |
| 24 | *C. pallida* | 11.07.2016 | Dresden | SN | *Apus apus* |
| 25 | *C. pallida* | 11.07.2016 | Dresden | SN | *Apus apus* |
| 26 | *C. pallida* | 11.07.2016 | Dresden | SN | *Apus apus* |
| 27 | *C. pallida* | 11.07.2016 | Dresden | SN | *Apus apus* |
| 28 | *C. pallida* | 11.07.2016 | Dresden | SN | *Apus apus* |
| 29 | *C. pallida* | 11.07.2016 | Dresden | SN | *Apus apus* |
| 30 | *C. pallida* | 11.07.2016 | Dresden | SN | *Apus apus* |
| 36 | *C. pallida* | 11.07.2016 | Dresden | SN | *Apus apus* |
| 37 | *C. pallida* | 12.07.2016 | Dresden | SN | *Apus apus* |
| 38 | *C. pallida* | 19.07.2016 | Dresden | SN | *Apus apus* |
| 2 | *C. pallida* | 23.07.2016 | Dresden | SN | *Apus apus* |
| 39 | *C. pallida* | 23.07.2016 | Dresden | SN | *Apus apus* |
| 40 | *C. pallida* | 23.07.2016 | Dresden | SN | *Apus apus* |
| 3 | *C. pallida* | 27.06.2018 | Rochlitz | SN | *Apus apus* |
| 4 | *C. pallida* | 27.06.2018 | Rochlitz | SN | *Apus apus* |
| 6 | *C. pallida* | 01.07.2018 | Rochlitz | SN | *Apus apus* |
| 7 | *C. pallida* | 01.07.2018 | Rochlitz | SN | *Apus apus* |
| 8 | *C. pallida* | 01.07.2018 | Rochlitz | SN | *Apus apus* |
| 9 | *C. pallida* | 01.07.2018 | Rochlitz | SN | *Apus apus* |
| JV-18-06 | *C. pallida* | 01.07.2018 | Rochlitz | SN | *Apus apus* |
| JV-18-06a | *C. pallida* | 01.07.2018 | Rochlitz | SN | *Apus apus* |
| JV-18-06b | *C. pallida* | 01.07.2018 | Rochlitz | SN | *Apus apus* |
| JV-18-06c | *C. pallida* | 01.07.2018 | Rochlitz | SN | *Apus apus* |
| JV-18-07 | *C. pallida* | 01.07.2018 | Rochlitz | SN | *Apus apus* |
| JV-18-07a | *C. pallida* | 01.07.2018 | Rochlitz | SN | *Apus apus* |
| JV-18-08 | *C. pallida* | 01.07.2018 | Rochlitz | SN | *Apus apus* |
| JV-18-08a | *C. pallida* | 01.07.2018 | Rochlitz | SN | *Apus apus* |
| JV-18-08b | *C. pallida* | 01.07.2018 | Rochlitz | SN | *Apus apus* |
| JV-18-09 | *C. pallida* | 01.07.2018 | Rochlitz | SN | *Apus apus* |
| JV-18-09a | *C. pallida* | 01.07.2018 | Rochlitz | SN | *Apus apus* |
| JV-18-10 | *C. pallida* | 01.07.2018 | Rochlitz | SN | *Apus apus* |
| JV-18-11 | *C. pallida* | 01.07.2018 | Rochlitz | SN | *Apus apus* |
| JV-18-11a | *C. pallida* | 01.07.2018 | Rochlitz | SN | *Apus apus* |
| JV-18-12 | *C. pallida* | 08.07.2018 | Rochlitz | SN | *Apus apus* |
| JV-18-12a | *C. pallida* | 08.07.2018 | Rochlitz | SN | *Apus apus* |
| JV-18-13 | *C. pallida* | 08.07.2018 | Rochlitz | SN | *Apus apus* |
| JV-18-14 | *C. pallida* | 08.07.2018 | Rochlitz | SN | *Apus apus* |
| JV-18-14a | *C. pallida* | 08.07.2018 | Rochlitz | SN | *Apus apus* |
| JV-18-15 | *C. pallida* | 08.07.2018 | Rochlitz | SN | *Apus apus* |
| JV-18-15a | *C. pallida* | 08.07.2018 | Rochlitz | SN | *Apus apus* |
| JV-18-15b | *C. pallida* | 08.07.2018 | Rochlitz | SN | *Apus apus* |
| MH-R-20-05 | *C. pallida* | 30.06.2020 | Dresden-Klotzsche | SN | *Apus apus* |
| MH-R-20-06 | *C. pallida* | 30.06.2020 | Dresden-Klotzsche | SN | *Apus apus* |
| MH-R-20-07 | *C. pallida* | 30.06.2020 | Dresden-Klotzsche | SN | *Apus apus* |
| MH-R-20-08 | *C. pallida* | 30.06.2020 | Dresden-Klotzsche | SN | *Apus apus* |
| MH-R-20-09 | *C. pallida* | 30.06.2020 | Dresden-Klotzsche | SN | *Apus apus* |
| MH-R-20-10 | *C. pallida* | 01.07.2020 | Dresden-Klotzsche | SN | *Apus apus* |
| FU-20-01 | *C. pallida* | 15.07.2020 | Bautzen | SN | *Apus apus* |
| FU-20-03 | *C. pallida* | 18.07.2020 | Bautzen | SN | *Apus apus* |
| JT-20-23 | *C. pallida* | 27.07.2020 | Chemnitz | SN | *Apus apus* |
| JT-20-23a | *C. pallida* | 27.07.2020 | Chemnitz | SN | *Apus apus* |
| JT-20-23b | *C. pallida* | 27.07.2020 | Chemnitz | SN | *Apus apus* |
| JV-18-02 | *C. pallida* | 27.07.2020 | Chemnitz | SN | *Apus apus* |
| JV-18-02a | *C. pallida* | 27.07.2020 | Chemnitz | SN | *Apus apus* |
| JV-18-02b | *C. pallida* | 27.07.2020 | Chemnitz | SN | *Apus apus* |
| JV-18-03 | *C. pallida* | 27.07.2020 | Chemnitz | SN | *Apus apus* |
| JV-18-03a | *C. pallida* | 27.07.2020 | Chemnitz | SN | *Apus apus* |
| MH-R-21-01 | *C. pallida* | 30.06.2021 | Dresden Lubminer Str. | SN | *Apus apus* |
| MH-R-21-01 | *C. pallida* | 30.06.2021 | Dresden Lubminer Str. | SN | *Apus apus* |
| MH-R-21-02 | *C. pallida* | 30.06.2021 | Dresden Lubminer Str. | SN | *Apus apus* |
| MH-R-21-03 | *C. pallida* | 30.06.2021 | Dresden Lubminer Str. | SN | *Apus apus* |
| MH-R-21-03 | *C. pallida* | 30.06.2021 | Dresden Lubminer Str. | SN | *Apus apus* |
| MH-R-21-04 | *C. pallida* | 30.06.2021 | Dresden Lubminer Str. | SN | *Apus apus* |
| MH-R-21-05 | *C. pallida* | 30.06.2021 | Dresden Lubminer Str. | SN | *Apus apus* |
| MH-R-21-05 | *C. pallida* | 30.06.2021 | Dresden Lubminer Str. | SN | *Apus apus* |
| MH-R-21-06 | *C. pallida* | 30.06.2021 | Dresden Lubminer Str. | SN | *Apus apus* |
| MH-R-21-06 | *C. pallida* | 30.06.2021 | Dresden Lubminer Str. | SN | *Apus apus* |
| MH-R-21-07 | *C. pallida* | 30.06.2021 | Dresden Lubminer Str. | SN | *Apus apus* |
| MH-R-21-07 | *C. pallida* | 30.06.2021 | Dresden Lubminer Str. | SN | *Apus apus* |
| MH-R-21-07a | *C. pallida* | 30.06.2021 | Dresden Lubminer Str. | SN | *Apus apus* |
| MH-R-21-08 | *C. pallida* | 30.06.2021 | Dresden Lubminer Str. | SN | *Apus apus* |
| MH-R-21-08 | *C. pallida* | 30.06.2021 | Dresden Lubminer Str. | SN | *Apus apus* |
| MH-R-21-08a | *C. pallida* | 30.06.2021 | Dresden Lubminer Str. | SN | *Apus apus* |
| MH-R-21-09 | *C. pallida* | 30.06.2021 | Dresden Lubminer Str. | SN | *Apus apus* |
| MH-R-21-09 | *C. pallida* | 30.06.2021 | Dresden Lubminer Str. | SN | *Apus apus* |
| MH-R-21-10 | *C. pallida* | 30.06.2021 | Dresden Lubminer Str. | SN | *Apus apus* |
| MH-R-21-10 | *C. pallida* | 30.06.2021 | Dresden Lubminer Str. | SN | *Apus apus* |
| MH-R-21-11 | *C. pallida* | 30.06.2021 | Dresden Lubminer Str. | SN | *Apus apus* |
| MH-R-21-11a | *C. pallida* | 30.06.2021 | Dresden Lubminer Str. | SN | *Apus apus* |
| MH-R-21-12 | *C. pallida* | 30.06.2021 | Dresden Lubminer Str. | SN | *Apus apus* |
| MH-R-21-12a | *C. pallida* | 30.06.2021 | Dresden Lubminer Str. | SN | *Apus apus* |
| MH-R-21-13 | *C. pallida* | 30.06.2021 | Dresden Lubminer Str. | SN | *Apus apus* |
| MH-R-21-14 | *C. pallida* | 30.06.2021 | Dresden Lubminer Str. | SN | *Apus apus* |
| MH-R-21-15 | *C. pallida* | 30.06.2021 | Dresden Lubminer Str. | SN | *Apus apus* |
| MH-R-21-16 | *C. pallida* | 30.06.2021 | Dresden Lubminer Str. | SN | *Apus apus* |
| MH-R-21-17 | *C. pallida* | 30.06.2021 | Dresden Lubminer Str. | SN | *Apus apus* |
| MH-R-21-18 | *C. pallida* | 30.06.2021 | Dresden Lubminer Str. | SN | *Apus apus* |
| MH-R-21-19 | *C. pallida* | 30.06.2021 | Dresden Lubminer Str. | SN | *Apus apus* |
| MH-R-21-20 | *C. pallida* | 30.06.2021 | Dresden Lubminer Str. | SN | *Apus apus* |
| MH-R-21-21 | *C. pallida* | 30.06.2021 | Dresden Lubminer Str. | SN | *Apus apus* |
| MH-R-21-22 | *C. pallida* | 30.06.2021 | Dresden Lubminer Str. | SN | *Apus apus* |
| MH-R-21-23 | *C. pallida* | 30.06.2021 | Dresden Lubminer Str. | SN | *Apus apus* |
| MH-R-21-24 | *C. pallida* | 30.06.2021 | Dresden Lubminer Str. | SN | *Apus apus* |
| MH-R-21-25 | *C. pallida* | 30.06.2021 | Dresden Lubminer Str. | SN | *Apus apus* |
| MH-R-21-26 | *C. pallida* | 30.06.2021 | Dresden Lubminer Str. | SN | *Apus apus* |
| MH-R-21-27 | *C. pallida* | 30.06.2021 | Dresden Lubminer Str. | SN | *Apus apus* |
| MH-R-21-28 | *C. pallida* | 30.06.2021 | Dresden Lubminer Str. | SN | *Apus apus* |
| MH-R-21-29 | *C. pallida* | 30.06.2021 | Dresden Lubminer Str. | SN | *Apus apus* |
| MH-R-21-30 | *C. pallida* | 30.06.2021 | Dresden Lubminer Str. | SN | *Apus apus* |
| MH-R-21-31 | *C. pallida* | 30.06.2021 | Dresden Lubminer Str. | SN | *Apus apus* |
| MH-R-21-32 | *C. pallida* | 30.06.2021 | Dresden Lubminer Str. | SN | *Apus apus* |
| MH-R-21-33 | *C. pallida* | 07.07.2021 | Dresden Lubminer Str. | SN | *Apus apus* |
| MH-R-21-34 | *C. pallida* | 07.07.2021 | Dresden Lubminer Str. | SN | *Apus apus* |
| MH-R-21-62 | *C. pallida* | 07.07.2021 | Dresden Lubminer Str. | SN | *Apus apus* |
| MH-R-21-63 | *C. pallida* | 07.07.2021 | Dresden Lubminer Str. | SN | *Apus apus* |
| MH-R-21-64 | *C. pallida* | 07.07.2021 | Dresden Lubminer Str. | SN | *Apus apus* |
| MH-R-21-65 | *C. pallida* | 07.07.2021 | Dresden Lubminer Str. | SN | *Apus apus* |
| MH-R-21-66 | *C. pallida* | 07.07.2021 | Dresden Lubminer Str. | SN | *Apus apus* |
| MH-R-21-67 | *C. pallida* | 07.07.2021 | Dresden Lubminer Str. | SN | *Apus apus* |
| MH-R-21-68 | *C. pallida* | 07.07.2021 | Dresden Lubminer Str. | SN | *Apus apus* |
| MH-R-21-69 | *C. pallida* | 07.07.2021 | Dresden Lubminer Str. | SN | *Apus apus* |
| MH-R-21-70 | *C. pallida* | 07.07.2021 | Dresden Lubminer Str. | SN | *Apus apus* |
| MH-R-21-71 | *C. pallida* | 07.07.2021 | Dresden Lubminer Str. | SN | *Apus apus* |
| MH-R-21-72 | *C. pallida* | 07.07.2021 | Dresden Lubminer Str. | SN | *Apus apus* |
| WN-20-05 | *O. avicularia* | 20.08.2021 | Holschaer Teich | SN | *Acrocephalus arundinaceus* |
| WN-20-06 | *O. avicularia* | 20.08.2021 | Holschaer Teich | SN | *Acrocephalus arundinaceus* |
| WN-20-07 | *O. avicularia* | 20.08.2021 | Holschaer Teich | SN | *Acrocephalus scirpaceus* |
| MH-R-20-04 | *O. avicularia* | 27.06.2020 | Lampertswalde | SN | *Athene noctua* |
| JT-20-04 | *O. avicularia* | 05.07.2020 | Gelenau Erzgebirge | SN | *Coccothraustes coccothraustes* |
| BM-R-21-02 | *O. avicularia* | 11.07.2021 | Grimma | SN | *Coccothraustes coccothraustes* |
| JT-22-10 | *O. avicularia* | 16.07.2022 | Gelenau - Hütte | SN | *Columba palumbus* |
| EH-18-18 | *O. avicularia* | 02.07.2018 | Bösdorf | SN | *Dendrocopos major* |
| BM-R-21-01 | *O. avicularia* | 03.08.2020 | Grimma | SN | *Dendrocopos major* |
| JT-R-21-01 | *O. avicularia* | 26.06.2021 | Gelenau | SN | *Dendrocopos major* |
| BM-R-21-03 | *O. avicularia* | 01.08.2021 | Grimma | SN | *Dendrocopos major* |
| JT-R-21-12 | *O. avicularia* | 22.08.2021 | Gelenau | SN | *Dendrocopos major* |
| EH-18-02 | *O. avicularia* | 18.05.2018 | Bösdorf | SN | *Emberiza citrinella* |
| JT-R-20-10 | *O. avicularia* | 25.07.2020 | Gelenau im Erzgebirge | SN | *Emberiza citrinella* |
| JT-22-15 | *O. avicularia* | 23.07.2022 | Gelenau - Hütte | SN | *Emberiza citrinella* |
| BM-18-02 | *O. avicularia* | 25.07.2018 | Hohnstädt Ruhenberg | SN | *Erithacus rubecula* |
| JT-20-06 | *O. avicularia* | 18.07.2020 | Gelenau Erzgebirge | SN | *Erithacus rubecula* |
| JT-R-20-13 | *O. avicularia* | 25.07.2020 | Gelenau im Erzgebirge | SN | *Erithacus rubecula* |
| JT-22-4 | *O. avicularia* | 02.07.2022 | Gelenau - Hütte | SN | *Erithacus rubecula* |
| JT-18-05 | *O. avicularia* | 14.06.2018 | Jahnsdorf Erzgebirge | SN | *Falco tinnunculus* |
| GE-21-04 | *O. avicularia* | 03.07.2021 | Leipzig | SN | *Falco tinnunculus* |
| GE-R-21-04 | *O. avicularia* | 20.07.2021 | Leipzig | SN | *Falco tinnunculus* |
| JT-22-7 | *O. avicularia* | 10.07.2022 | A-Dorf | SN | *Falco tinnunculus* |
| EH-18-01 | *O. avicularia* | 18.05.2018 | Elsterstausee Bösdorf | SN | *Garrulus glandarius* |
| MF-20-12 | *O. avicularia* | 02.07.2020 | Welschhufe | SN | *Hirundo rustica* |
| JT-20-07 | *O. avicularia* | 19.07.2020 | Gelenau Erzgebirge | SN | *Parus major* |
| JT-22-8 | *O. avicularia* | 16.07.2022 | Gelenau - Hütte | SN | *Parus major* |
| EH-18-04 | *O. avicularia* | 03.06.2018 | Bösdorf | SN | *Passer domesticus* |
| JT-R-21-02 | *O. avicularia* | 03.07.2021 | Gelenau | SN | *Phoenicurus ochruros* |
| JT-20-02 | *O. avicularia* | 24.06.2020 | Gelenau Erzgebirge | SN | *Phylloscopus collybita* |
| JT-20-01 | *O. avicularia* | 13.06.2020 | Gelenau Erzgebirge | SN | *Pyrrhula pyrrhula* |
| JT-22-6 | *O. avicularia* | 02.07.2022 | Gelenau - Hütte | SN | *Sitta europaea* |
| JT-R-20-15 | *O. avicularia* | 25.07.2020 | Gelenau im Erzgebirge | SN | *Sylvia atricapilla* |
| JT-R-21-04 | *O. avicularia* | 18.07.2021 | Gelenau | SN | *Sylvia atricapilla* |
| JT-22-1 | *O. avicularia* | 25.06.2022 | Gelenau - Hütte | SN | *Sylvia atricapilla* |
| JT-R-20-12 | *O. avicularia* | 25.07.2020 | Gelenau im Erzgebirge | SN | *Sylvia borin* |
| CG-R-20-13 | *O. avicularia* | 18.08.2020 | Possendorf | SN | *Sylvia borin* |
| BM-18-01 | *O. avicularia* | 21.05.2018 | Hohnstädt Ruhenberg | SN | *Turdus merula* |
| JT-18-02 | *O. avicularia* | 02.06.2018 | Gelenau Erzgebirge | SN | *Turdus merula* |
| EH-18-03 | *O. avicularia* | 03.06.2018 | Bösdorf | SN | *Turdus merula* |
| JT-18-07 | *O. avicularia* | 16.06.2018 | Gelenau Erzgebirge | SN | *Turdus merula* |
| EH-18-17 | *O. avicularia* | 02.07.2018 | Bösdorf | SN | *Turdus merula* |
| JT-18-15 | *O. avicularia* | 21.07.2018 | Gelenau Erzgebirge | SN | *Turdus merula* |
| JT-18-16 | *O. avicularia* | 21.07.2018 | Gelenau Erzgebirge | SN | *Turdus merula* |
| JT-18-21 | *O. avicularia* | 18.08.2018 | Gelenau Erzgebirge | SN | *Turdus merula* |
| JT-18-22 | *O. avicularia* | 18.08.2018 | Gelenau Erzgebirge | SN | *Turdus merula* |
| JT-20-08 | *O. avicularia* | 19.07.2020 | Gelenau Erzgebirge | SN | *Turdus merula* |
| CG-R-20-14 | *O. avicularia* | 23.08.2020 | Possendorf | SN | *Turdus merula* |
| CG-R-20-16 | *O. avicularia* | 03.09.2020 | Possendorf | SN | *Turdus merula* |
| CG-R-20-17 | *O. avicularia* | 09.09.2020 | Possendorf | SN | *Turdus merula* |
| JT-R-21-03 | *O. avicularia* | 03.07.2021 | Gelenau | SN | *Turdus merula* |
| JT-R-21-05 | *O. avicularia* | 18.07.2021 | Gelenau | SN | *Turdus merula* |
| WN-20-04 | *O. avicularia* | 20.08.2021 | Holschaer Teich | SN | *Turdus merula* |
| JT-22-2 | *O. avicularia* | 25.06.2022 | Gelenau - Hütte | SN | *Turdus merula* |
| JT-22-2a | *O. avicularia* | 25.06.2022 | Gelenau - Hütte | SN | *Turdus merula* |
| JT-22-5 | *O. avicularia* | 02.07.2022 | Gelenau - Hütte | SN | *Turdus merula* |
| JT-22-5 | *O. avicularia* | 02.07.2022 | Gelenau - Hütte | SN | *Turdus merula* |
| JT-22-5a | *O. avicularia* | 02.07.2022 | Gelenau - Hütte | SN | *Turdus merula* |
| JT-22-11 | *O. avicularia* | 23.07.2022 | Gelenau - Hütte | SN | *Turdus merula* |
| JT-22-12 | *O. avicularia* | 23.07.2022 | Gelenau - Hütte | SN | *Turdus merula* |
| JT-18-01 | *O. avicularia* | 27.05.2018 | Gelenau Erzgebirge | SN | *Turdus philomelos* |
| EH-18-14 | *O. avicularia* | 11.06.2018 | Bösdorf | SN | *Turdus philomelos* |
| JT-18-13 | *O. avicularia* | 15.07.2018 | Gelenau Erzgebirge | SN | *Turdus philomelos* |
| JT-18-24 | *O. avicularia* | 18.08.2018 | Gelenau Erzgebirge | SN | *Turdus philomelos* |
| JT-20-31 | *O. avicularia* | 11.10.2020 | Gelenau Erzgebirge | SN | *Turdus philomelos* |
| JT-22-13 | *O. avicularia* | 23.07.2022 | Gelenau - Hütte | SN | *Turdus philomelos* |
| JT-22-13a | *O. avicularia* | 23.07.2022 | Gelenau - Hütte | SN | *Turdus philomelos* |
| JT-22-14 | *O. avicularia* | 23.07.2022 | Gelenau - Hütte | SN | *Turdus philomelos* |
| JT-22-14a | *O. avicularia* | 23.07.2022 | Gelenau - Hütte | SN | *Turdus philomelos* |
| EH-18-05 | *O. biloba* | 04.06.2018 | Gundorf | SN | *Hirundo rustica* |
| EH-18-06 | *O. biloba* | 04.06.2018 | Gundorf | SN | *Hirundo rustica* |
| EH-18-08 | *O. biloba* | 04.06.2018 | Gundorf | SN | *Hirundo rustica* |
| EH-18-09 | *O. biloba* | 04.06.2018 | Gundorf | SN | *Hirundo rustica* |
| EH-18-10 | *O. biloba* | 04.06.2018 | Gundorf | SN | *Hirundo rustica* |
| EH-18-11 | *O. biloba* | 04.06.2018 | Gundorf | SN | *Hirundo rustica* |
| EH-18-13 | *O. biloba* | 09.06.2018 | Zoo Leipzig | SN | *Hirundo rustica* |
| EH-18-15 | *O. biloba* | 18.06.2018 | Leipzig, Wildpark bei Connewitz | SN | *Hirundo rustica* |
| EH-18-16 | *O. biloba* | 22.06.2018 | Gundorf | SN | *Hirundo rustica* |
| EH-18-20 | *O. biloba* | 22.07.2018 | Zoo Leipzig | SN | *Hirundo rustica* |
| EH-18-21 | *O. biloba* | 23.07.2018 | Gundorf | SN | *Hirundo rustica* |
| EH-18-23 | *O. biloba* | 23.07.2018 | Gundorf | SN | *Hirundo rustica* |
| EH-18-24 | *O. biloba* | 23.07.2018 | Gundorf | SN | *Hirundo rustica* |
| EH-18-25 | *O. biloba* | 23.07.2018 | Gundorf | SN | *Hirundo rustica* |
| TB-R-20-07 | *O. biloba* | 01.06.2020 | Streitwald | SN | *Hirundo rustica* |
| TB-R-20-09 | *O. biloba* | 02.06.2020 | Wenigossa | SN | *Hirundo rustica* |
| TB-R-20-10 | *O. biloba* | 02.06.2020 | Wenigossa | SN | *Hirundo rustica* |
| MH-R-20-01 | *O. biloba* | 21.06.2020 | Obernaundorf | SN | *Hirundo rustica* |
| MH-R-20-02 | *O. biloba* | 22.06.2020 | Bannewitz | SN | *Hirundo rustica* |
| GE-R-21-01 | *O. biloba* | 10.06.2021 | Taucha | SN | *Hirundo rustica* |
| GE-R-21-02 | *O. biloba* | 10.06.2021 | Taucha | SN | *Hirundo rustica* |
| GE-R-21-03 | *O. biloba* | 10.06.2021 | Taucha | SN | *Hirundo rustica* |
| GE-R-21-05 | *O. biloba* | 31.07.2021 | Taucha | SN | *Hirundo rustica* |
| JT-20-30 | *O. chloropus* | 04.10.2020 | Gelenau Erzgebirge | SN | *Parus major* |
| JT-18-30 | *O. fringillina* | 07.10.2018 | Gelenau Erzgebirge | SN | *Cyanistes caeruleus* |
| JT-20-32 | *O. fringillina* | 15.11.2020 | Gelenau Erzgebirge | SN | *Cyanistes caeruleus* |
| JT-18-09 | *O. fringillina* | 23.06.2018 | Gelenau Erzgebirge | SN | *Erithacus rubecula* |
| JT-18-18 | *O. fringillina* | 04.08.2018 | Gelenau Erzgebirge | SN | *Erithacus rubecula* |
| JT-20-09 | *O. fringillina* | 19.07.2020 | Gelenau Erzgebirge | SN | *Erithacus rubecula* |
| JT-R-20-16 | *O. fringillina* | 25.07.2020 | Gelenau im Erzgebirge | SN | *Erithacus rubecula* |
| JT-20-18 | *O. fringillina* | 16.08.2020 | Gelenau Erzgebirge | SN | *Erithacus rubecula* |
| JT-20-26 | *O. fringillina* | 29.08.2020 | Gelenau Erzgebirge | SN | *Erithacus rubecula* |
| JT-R-21-10 | *O. fringillina* | 14.08.2021 | Gelenau | SN | *Erithacus rubecula* |
| JT-22-16 | *O. fringillina* | 23.07.2022 | Gelenau - Hütte | SN | *Erithacus rubecula* |
| EH-18-19 | *O. fringillina* | 14.07.2018 | Bösdorf | SN | *Lanius collurio* |
| JT-R-21-07 | *O. fringillina* | 31.07.2021 | Gelenau | SN | *Phylloscopus collybita* |
| JT-R-21-08 | *O. fringillina* | 14.08.2021 | Gelenau | SN | *Phylloscopus collybita* |
| JT-18-27 | *O. fringillina* | 16.09.2018 | Gelenau Erzgebirge | SN | *Poecile montanus* |
| JT-20-21 | *O. fringillina* | 16.08.2020 | Gelenau Erzgebirge | SN | *Poecile montanus* |
| JT-20-24 | *O. fringillina* | 22.08.2020 | Gelenau Erzgebirge | SN | *Poecile palustris* |
| JT-20-27 | *O. fringillina* | 05.09.2020 | Gelenau Erzgebirge | SN | *Pyrrhula pyrrhula* |
| JT-20-29 | *O. fringillina* | 04.10.2020 | Gelenau Erzgebirge | SN | *Regulus ignicapilla* |
| JT-R-21-06 | *O. fringillina* | 24.07.2021 | Gelenau | SN | *Regulus ignicapilla* |
| JT-R-21-09 | *O. fringillina* | 14.08.2021 | Gelenau | SN | *Regulus ignicapilla* |
| JT-18-11 | *O. fringillina* | 01.07.2018 | Gelenau Erzgebirge | SN | *Sylvia atricapilla* |
| JT-18-14 | *O. fringillina* | 15.07.2018 | Gelenau Erzgebirge | SN | *Sylvia atricapilla* |
| JT-18-19 | *O. fringillina* | 12.08.2018 | Gelenau Erzgebirge | SN | *Sylvia atricapilla* |
| JT-18-23 | *O. fringillina* | 18.08.2018 | Gelenau Erzgebirge | SN | *Sylvia atricapilla* |
| JT-R-20-14 | *O. fringillina* | 25.07.2020 | Gelenau im Erzgebirge | SN | *Sylvia atricapilla* |
| JT-20-28 | *O. fringillina* | 05.09.2020 | Gelenau Erzgebirge | SN | *Sylvia atricapilla* |
| JT-R-21-11 | *O. fringillina* | 14.08.2021 | Gelenau | SN | *Sylvia atricapilla* |
| SF-R-20-02 | *C. hirundinis* | 15.07.2020 | Steckby | ST | *Delichon urbicum* |
| SF-R-20-03 | *C. hirundinis* | 15.07.2020 | Steckby | ST | *Delichon urbicum* |
| SF-R-20-04 | *C. hirundinis* | 15.07.2020 | Steckby | ST | *Delichon urbicum* |
| SF-R-20-05 | *C. hirundinis* | 17.07.2020 | Steckby | ST | *Delichon urbicum* |
| SF-R-20-06 | *C. hirundinis* | 17.07.2020 | Steckby | ST | *Delichon urbicum* |
| SF-R-20-07 | *C. hirundinis* | 17.07.2020 | Steckby | ST | *Delichon urbicum* |
| SF-R-20-08 | *C. hirundinis* | 17.07.2020 | Steckby | ST | *Delichon urbicum* |
| SF-R-20-09 | *C. hirundinis* | 17.07.2020 | Steckby | ST | *Delichon urbicum* |
| SF-R-20-10 | *C. hirundinis* | 17.07.2020 | Steckby | ST | *Delichon urbicum* |
| MF-20-10 | *O. avicularia* | 14.07.2021 | Halberstadt | ST | *Falco tinnunculus* |
| MS-R-21-02 | *O. avicularia* | 05.07.2021 | Hakel | ST | *Milvus milvus* |
| MS-R-21-02 | *O. avicularia* | 05.07.2021 | Hakel | ST | *Milvus milvus* |
| MS-R-21-03 | *O. avicularia* | 06.07.2021 | Hakel | ST | *Milvus milvus* |
| MS-R-21-05 | *O. avicularia* | 14.07.2021 | Hakel | ST | *Milvus milvus* |
| MS-R-21-06 | *O. avicularia* | 15.07.2021 | Hakel | ST | *Milvus milvus* |
| MF-20-13 | *O. avicularia* | 14.07.2021 | Halberstadt | ST | *unknown* |
| MF-20-13b | *O. avicularia* | 14.07.2021 | Halberstadt | ST | *unknown* |
| MF-20-13c | *O. avicularia* | 14.07.2021 | Halberstadt | ST | *unknown* |
| MF-20-13d | *O. avicularia* | 14.07.2021 | Halberstadt | ST | *unknown* |
| MF-20-13e | *O. avicularia* | 14.07.2021 | Halberstadt | ST | *unknown* |
| MF-20-13f | *O. avicularia* | 14.07.2021 | Halberstadt | ST | *unknown* |
| MF-20-13g | *O. avicularia* | 14.07.2021 | Halberstadt | ST | *unknown* |
| MF-20-13h | *O. avicularia* | 14.07.2021 | Halberstadt | ST | *unknown* |
| MF-20-13i | *O. avicularia* | 14.07.2021 | Halberstadt | ST | *unknown* |
| TB-R-20-01 | *O. biloba* | 01.06.2020 | Benndorf | ST | *Hirundo rustica* |
| TB-R-20-05 | *O. biloba* | 01.06.2020 | Benndorf | ST | *Hirundo rustica* |
| TB-R-20-06 | *O. biloba* | 01.06.2020 | Benndorf | ST | *Hirundo rustica* |
| AG-20-41 | *O. fringillina* | 16.09.2021 | Numburg | ST | *Sylvia atricapilla* |
| AG-20-42 | *O. fringillina* | 16.09.2021 | Numburg | ST | *Sylvia atricapilla* |
| AG-20-43 | *O. fringillina* | 16.09.2021 | Numburg | ST | *Sylvia atricapilla* |
| KE-21-06 | *O. avicularia* | 14.08.2021 | Elmshorn | SH | *Columba livia f*. *domestica* |
| JW-21-01 | *O. avicularia* | 20.06.2021 | Bornhöved | SH | *Dendrocopos major* |
| JW-21-02 | *O. avicularia* | 20.07.2021 | Neumünster | SH | *Falco tinnunculus* |
| EH-19-03 | *O. avicularia* | 15.07.2019 | Bösdorf | SH | *Jynx torquilla* |
| EH-19-04 | *O. avicularia* | 20.07.2019 | Bösdorf | SH | *Jynx torquilla* |
| EH-19-01 | *O. avicularia* | 29.06.2019 | Bösdorf | SH | *Lanius collurio* |
| JW-21-03 | *O. avicularia* | 20.07.2021 | Schmalfeld | SH | *Phasianus colchicus* |
| EH-19-05 | *O. avicularia* | 20.07.2019 | Bösdorf | SH | *Sylvia nisoria* |
| KE-21-01 | *O. avicularia* | 03.08.2021 | Elmshorn | SH | *Turdus merula* |
| KE-21-03 | *O. avicularia* | 08.08.2021 | Elmshorn | SH | *Turdus merula* |
| KE-21-04 | *O. avicularia* | 11.08.2021 | Norderstedt | SH | *Turdus merula* |
| KE-21-05 | *O. avicularia* | 14.08.2021 | Norderstedt | SH | *Turdus merula* |
| KE-21-07 | *O. avicularia* | 14.08.2021 | Schenefeld | SH | *Turdus merula* |
| KE-21-08 | *O. avicularia* | 18.08.2021 | Elmshorn | SH | *Turdus merula* |
| KE-21-02 | *O. avicularia* | 05.08.2021 | Elmshorn | SH | *unknown* |
| KE-21-09 | *O. avicularia* | 29.08.2021 | Elmshorn | SH | *unknown* |
| GO-20-5 | *O. fringillina* | 11.08.2020 | Greifswalder Oie | SH | *Parus major* |
| GO-20-8 | *O. fringillina* | 26.08.2020 | Greifswalder Oie | SH | *Parus major* |
| AG-20-38 | *O. avicularia* | 24.07.2021 | Reifenstein | TH | *Acrocephalus palustris* |
| AG-20-15 | *O. avicularia* | 09.08.2020 | Reifenstein | TH | *Carduelis carduelis* |
| AG-R-21-02 | *O. avicularia* | 06.08.2021 | Reifenstein | TH | *Columba palumbus* |
| AG-R-21-05 | *O. avicularia* | 27.08.2021 | Wachstedt | TH | *Dendrocopos major* |
| AG-20-35 | *O. avicularia* | 18.07.2021 | Wachstedt | TH | *Fringilla coelebs* |
| AG-20-37 | *O. avicularia* | 18.07.2021 | Wachstedt | TH | *Fringilla coelebs* |
| AG-20-39 | *O. avicularia* | 24.07.2021 | Reifenstein | TH | *Lanius collurio* |
| AG-20-16 | *O. avicularia* | 09.08.2020 | Reifenstein | TH | *Passer montanus* |
| AG-20-40 | *O. avicularia* | 24.07.2021 | Reifenstein | TH | *Passer montanus* |
| AG-R-20-06 | *O. avicularia* | 02.08.2020 | Reifenstein | TH | *Sylvia atricapilla* |
| AG-20-01 | *O. avicularia* | 20.07.2020 | Reifenstein | TH | *Turdus merula* |
| AG-R-20-02 | *O. avicularia* | 28.07.2020 | Kleinbartlott | TH | *Turdus merula* |
| AG-R-20-03 | *O. avicularia* | 30.07.2020 | Reifenstein | TH | *Turdus merula* |
| AG-R-20-03a | *O. avicularia* | 30.07.2020 | Reifenstein | TH | *Turdus merula* |
| AG-20-27 | *O. avicularia* | 06.09.2020 | Reifenstein | TH | *Turdus merula* |
| AG-20-34 | *O. avicularia* | 17.07.2021 | Reifenstein | TH | *Turdus merula* |
| AG-R-20-04 | *O. avicularia* | 30.07.2020 | Reifenstein | TH | *Turdus philomelos* |
| AG-20-12 | *O. avicularia* | 07.08.2020 | Reifenstein | TH | *Turdus philomelos* |
| AG-20-13 | *O. avicularia* | 09.08.2020 | Reifenstein | TH | *Turdus philomelos* |
| AG-20-25 | *O. avicularia* | 23.08.2020 | Reifenstein | TH | *Turdus philomelos* |
| AG-20-33 | *O. avicularia* | 17.07.2021 | Reifenstein | TH | *Turdus philomelos* |
| AG-R-21-04 | *O. avicularia* | 22.08.2021 | Reifenstein | TH | *Turdus philomelos* |
| AG-R-21-03 | *O. fringillina* | 14.08.2021 | Reifenstein | TH | Acrocephalus palustris |
| AG-20-14 | *O. fringillina* | 09.08.2020 | Reifenstein | TH | *Acrocephalus scirpaceus* |
| AG-R-21-07 | *O. fringillina* | 10.09.2021 | Numburg | TH | *Acrocephalus scirpaceus* |
| BM-R-21-04 | *O. fringillina* | 12.09.2021 | Numburg | TH | *Acrocephalus scirpaceus* |
| BM-R-21-09 | *O. fringillina* | 15.09.2021 | Numburg | TH | *Acrocephalus scirpaceus* |
| AG-R-21-08 | *O. fringillina* | 11.09.2021 | Numburg | TH | *Emberiza citrinella* |
| AG-R-21-01 | *O. fringillina* | 30.07.2021 | Wachstedt | TH | *Erithacus rubecula* |
| BM-R-21-10 | *O. fringillina* | 16.09.2021 | Numburg | TH | *Erithacus rubecula* |
| BM-R-21-05 | *O. fringillina* | 12.09.2021 | Numburg | TH | *Motacilla cinerea* |
| AG-R-21-06 | *O. fringillina* | 27.08.2021 | Wachstedt | TH | *Parus major* |
| BM-R-21-08 | *O. fringillina* | 14.09.2021 | Numburg | TH | *Parus major* |
| BM-R-21-06 | *O. fringillina* | 12.09.2021 | Numburg | TH | *Phylloscopus collybita* |
| AG-20-29 | *O. fringillina* | 27.09.2020 | Wachstedt | TH | *Poecile palustris* |
| AG-R-20-08 | *O. fringillina* | 04.08.2020 | Reifenstein | TH | *Sylvia atricapilla* |
| AG-20-28 | *O. fringillina* | 06.09.2020 | Reifenstein | TH | *Sylvia atricapilla* |
| BM-R-21-07 | *O. fringillina* | 13.09.2021 | Numburg | TH | *Sylvia atricapilla* |
| AG-R-20-09 | *O. fringillina* | 04.08.2020 | Reifenstein | TH | *Sylvia borin* |
| AG-R-20-05 | *O. fringillina* | 02.08.2020 | Reifenstein | TH | *Turdus philomelos* |
| AG-R-21-09 | *O. fringillina* | 11.09.2021 | Numburg | TH | *unknown* |
| AG-R-21-10 | *O. fringillina* | 12.09.2021 | Numburg | TH | *unknown* |

^1^*Crataerina (C.) pallida*, *Ornithomya (O.) avicularia, Ornithomya (O.) biloba, Ornithomya (O.) fringillina, Ornithophila (O.) metallica, Crataerina (C.) hirundinis, Pseudolynchia (P.) canariensis, Ornithomya (O.) chloropus*

^2^FS-Federal State: BW-Baden-Württemberg, BY-Bavaria, BB-Brandenburg, HE-Hesse, MV- Mecklenburg-Western Pomerania, NI- Lower Saxony, NW- North Rhine-Westphalia, RP- Rhineland-Palatinate, SL-Saarland, SN-Saxony, ST- Saxony-Anhalt, SH-Schleswig-Holstein, TH-Thuringia
